# Expression of Human β3GalT5–1 in Insect Cells as Active Glycoforms for the Efficient Synthesis of Cancer-Associated Globo-Series Glycans

**DOI:** 10.1021/jacs.4c11723

**Published:** 2025-03-25

**Authors:** Chih-Chuan Kung, Jennifer M. Lo, Kuo-Shiang Liao, Chung-Yi Wu, Li-Chun Cheng, Cinya Chung, Tsui-Ling Hsu, Che Ma, Chi-Huey Wong

**Affiliations:** † Genomics Research Center, Academia Sinica, Taipei 11529, Taiwan; ‡ Department of Chemistry, The Scripps Research Institute, La Jolla, California 92037, United States

## Abstract

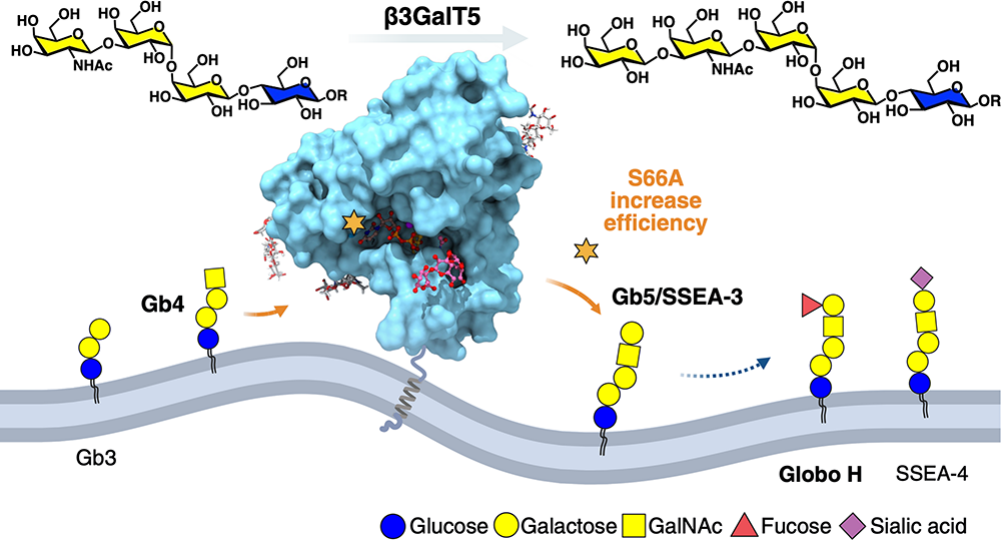

The globo-series glycosphingolipids (GSLs) are unique glycolipids exclusively expressed on the cell surface of various types of cancer and have been used as targets for the development of cancer vaccines and therapeutics. A practical enzymatic method has been developed for the synthesis of globo-series glycans, where the conversion of Gb4 to Gb5 (SSEA-3) glycan based on the microbial galactosyltransferase LgtD is relatively inefficient compared to other steps. To improve the efficiency, we explored the two human galactosyltransferase (β3GalT5) isozymes in cancer cells for this reaction and found that isozyme 1 (β3GalT5–1) is more active than isozyme 2 (β3GalT5–2). We then identified a common soluble domain of the two β3GalT5 isozymes as a candidate and evaluated the activity and substrate specificity of the glycosylated and nonglycosylated glycoforms. The glycoforms expressed in Sf9 cells were selected, and a site-specific alanine scan was performed to identify S66A β3GalT5 variant with 10-fold more efficiency than LgtD for the synthesis of globo-series glycans. The X-ray structure of β3GalT5–1 was determined for molecular modeling, and the result together with kinetic data were used to rationalize the improvement in catalysis.

## Introduction

The globo-series glycosphingolipids (GSLs), including stage-specific embryonic antigen-3 (SSEA-3), SSEA-4, and Globo-H, are often found with irregular expression patterns on the cell surface of various types of cancer^1,2^ to promote cancer progression.^3−5^ The expression levels of globo-series glycans in clinical samples correlate with tumor metastasis and progression, as well as poor survival.^6−12^ These tumor-associated carbohydrate antigens^13^ have been considered promising targets for the development of new therapies, especially cancer vaccines.^6−22^

**Figure 1:**
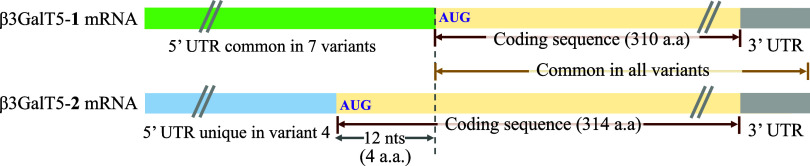
RNA transcripts of β3GalT5–1 and β3GalT5–2. The yellow boxes represent the full-length translational sequence of β3GalT5–1 (M1-V310) including the soluble domain (N29–V310), and an additional 4 amino acids in the N-terminal of β3GalT5–2 (314 amino acids). The green box with 140 nucleotides before the start codon AUG is a common sequence of β3GalT5–1 in the seven of eight transcripts. The blue box represents the unique sequence in one variant (variant 4) for β3GalT5–2.

In the biosynthesis of globo-series GSLs, the enzyme β1,3-galactosyltransferase 5 (β3GalT5) expressed in cancer cells catalyzes the rate-limiting conversion of Gb4 to Gb5 (SSEA-3), which is subsequently transformed to Globo-H and SSEA-4 by fucosyltransferase 2 (FUT2) and sialyltransferase (ST3Gal1), respectively. β3GalT5 exists as two distinct isozymes (β3GalT5–1 and β3GalT5–2) with 310 amino acids for β3GalT5–1 and an additional four amino acids in the N-terminal of β3GalT5–2 (Figure 1). Knockdown of both isozymes simultaneously in MDA-MB-231 breast cancer cells using shRNA was shown to inhibit proliferation and induce cancer cell apoptosis while there was no effect on normal cells.^19^ However, the expression of these two isozymes in normal and cancer cells and their corresponding functions remain unclear. Nevertheless, the cancer vaccine with Globo-H glycan conjugated to keyhole limpet hemocyanin (KLH) and adjuvanted with QS-21 has been advanced to phase 3 clinical trials for the treatment of Globo-H positive patients with early stage triple-negative breast cancer (NCT03562637).^23,24^ Another cancer vaccine with Globo-H glycan conjugated to Crim197 and adjuvanted with C34, a glycolipid adjuvant designed to induce class switch, has been advanced to phase 2 trials for the treatment of Globo-H positive patients with tyrosine kinase inhibitor (TKI)-resistant nonsmall cell lung cancer (NCT05442060) and esophageal cancer (NCT05376423).

To enable the late-stage clinical study, we developed a practical enzymatic method coupled with sugar nucleotide regeneration for the synthesis of Globo-H glycan.^25^ However, the microbial enzyme β1,3-*N*-acetylgalactosaminyltransferase (LgtD) used for the synthesis of Gb5 glycan from Gb4 glycan was not efficient. In this study, we explored the synthetic utility of human β3GalT5 to improve the process. Since β3GalT5 is a membrane-bound glycoprotein and exists as two isozymes, we first designed specific probes and dicer-substrate siRNA to knockdown specific isozymes in breast cancer cells and found that the expression level and activity of β3GalT5–1 were higher than those of β3GalT5–2. We also investigated the impact of glycosylation on protein folding and activity and found that the soluble domain of β3GalT5–1 (residues N29–V310) expressed in insect cells is an efficient catalyst for synthesis. In addition, the X-ray structure and substrate specificity of soluble β3GalT5–1 were determined, and a site-specific alanine scan was performed to identify the S66A β3GalT5–1 variant with at least 10-fold improvement in activity for the efficient synthesis of globo-series glycans

## Results and Discussion

### Expression of β3GalT5–1 and β3GalT5–2 in Normal and Cancer Cells

Comparing the two isozymes of human β3GalT5 (Figure 1), β3GalT5–1 is composed of 310 amino acids, residues 1–7 located in the cytosol, residues 8–28 in the transmembrane domain, and residues 29–310 in the soluble Golgi luminal domain. β3GalT5–2 contains the same 310-amino acid sequence as β3GalT5–1 with an additional four amino acids at the N-terminal. At the RNA level (Figure S1), eight variants of human β3GalT5 exist, seven of which encode the β3GalT5–1 protein and one variant (variant 4, NM_033172.3) encoding the β3GalT5–2 protein. By aligning all transcript variants, we found that except variant 4, the seven variants encoding the β3GalT5–1 protein have the same 140 nucleotide sequences in the 5′ UTR of β3GalT5–1. Therefore, we designed specific PCR TaqMan probes to detect the expression of β3GalT5–1 and β3GalT5–2 (Figure 1) in different cells including breast, colon, and lung cancer cells. We also employed droplet-digital PCR (ddPCR) to quantify the RNA levels of β3GalT5 isozymes (Figure S1B) and found that cancer cells contained higher copy numbers than normal cells and the lung cancer cell line CL-5 with greater progression ability than CL-0 exhibited a higher level of β3GalT5–1 expression. Conversely, β3GalT5–2 was expressed at extremely low levels in most normal and cancer cells, with slightly higher expression in the breast cancer cell line MDA-MB231 and colon cancer cell lines SW1116 and colo205 (Figure S1C).

### Specific Gene Suppression of β3GalT5 Isozymes in Breast Cancer Cells

To investigate the functions of β3GalT5–1 and β3GalT5–2 in breast cancer cells, we first designed and screened 11 dicer-substrate siRNAs (dsiRNAs) based on the common 5′ UTR of β3GalT5. To specifically target β3GalT5–1 or β3GalT5–2 in breast cancer cells, we chose MDA-MB231 as a model because both β3GalT5–1 and β3GalT5–2 are expressed in this cell line (Figure S1D). Then, we evaluated the knockdown efficiency of β3GalT5–1 among 11 dsiRNA in MDA-MB-231 cells. Of all knockdown studies 48 h after electroporation, #4 dsiRNA was the most efficient in inhibiting the expression of β3GalT5–1 (Figure S2A). In addition, we evaluated the knockdown efficiency at different time points; the percentage of *β3GalT5–1* knockdown within 84 h was about 70% and continued to increase until 144 h (Figure S2B). Next, we analyzed the cell surface levels of SSEA-3, SSEA-4, and Globo-H by flow cytometry at different time points after the knockdown of β3GalT5–1 and found that the expression of SSEA-3 and Globo-H was significantly reduced in MDA-MB231 cells, while SSEA-4 expression showed little change from 48 to 96 h (Figure S2C), perhaps due to the remaining 20% activity of β3GalT5–1 and β3GalT5–2. We also examined the β3GalT5–1-catalyzed glycosylation in MCF-7 cells due to the higher expression of β3GalT5–1, SSEA-3, and Globo-H and lower expression of β3GalT5–2 in this cell line compared to MDA-MB231 (Figures S1C and S2D). We found an increased expression of Gb4 and a decreased expression of Globo-H in MCF-7 cells with *β3GalT5–1* knockdown as measured by mass spectrometry (MS) analysis (Figure S2E), while the surface levels of SSEA-3, SSEA-4, and Globo-H decreased based on flow cytometry analysis (Figure S2D). For the *O*-glycans, it is known that β3GalT5 is involved in core 3 and core 4 extension. After the knockdown of *β3GalT5–1*, the core 1-related glycans decreased while the core 2- and 3-related glycans increased, probably due to the disruption of core 3 extension (Figure S2F). This phenomenon is similar to the inhibition of breast cancer development in mice caused by the absence of core 1-derived mucin-type *O*-glycosylation.^26,27^ Additionally, core 3 *O*-glycan structures are known to decrease in cancer cells.^28,29^ However, recent findings demonstrated that, following the knockout of β3GalT5 in mice, the glycosylation levels of core 1 and 2 increased, while that of core 3 and 4 decreased in colonic MUC2.^30^ These contrasting results may be attributed to the differences in cell types between normal and cancerous conditions.

In addition, the percentage of mannose-type *N*-glycan decreased, while the complexed-type glycans including biantennary, triantennary, and tetra-antennary *N*-glycans increased (Figure S2G); a similar change was found in previous studies.^31,32^ However, the knockdown of *β3GalT5–2*, which was expressed at an extremely low level in MCF-7, showed no effect on SSEA-3/SSEA-4/Globo-H expression as observed in flow cytometry.

### Purification of Membrane-Bound β3GalT5 Isozymes with Detergent LMNG

**Figure 2:**
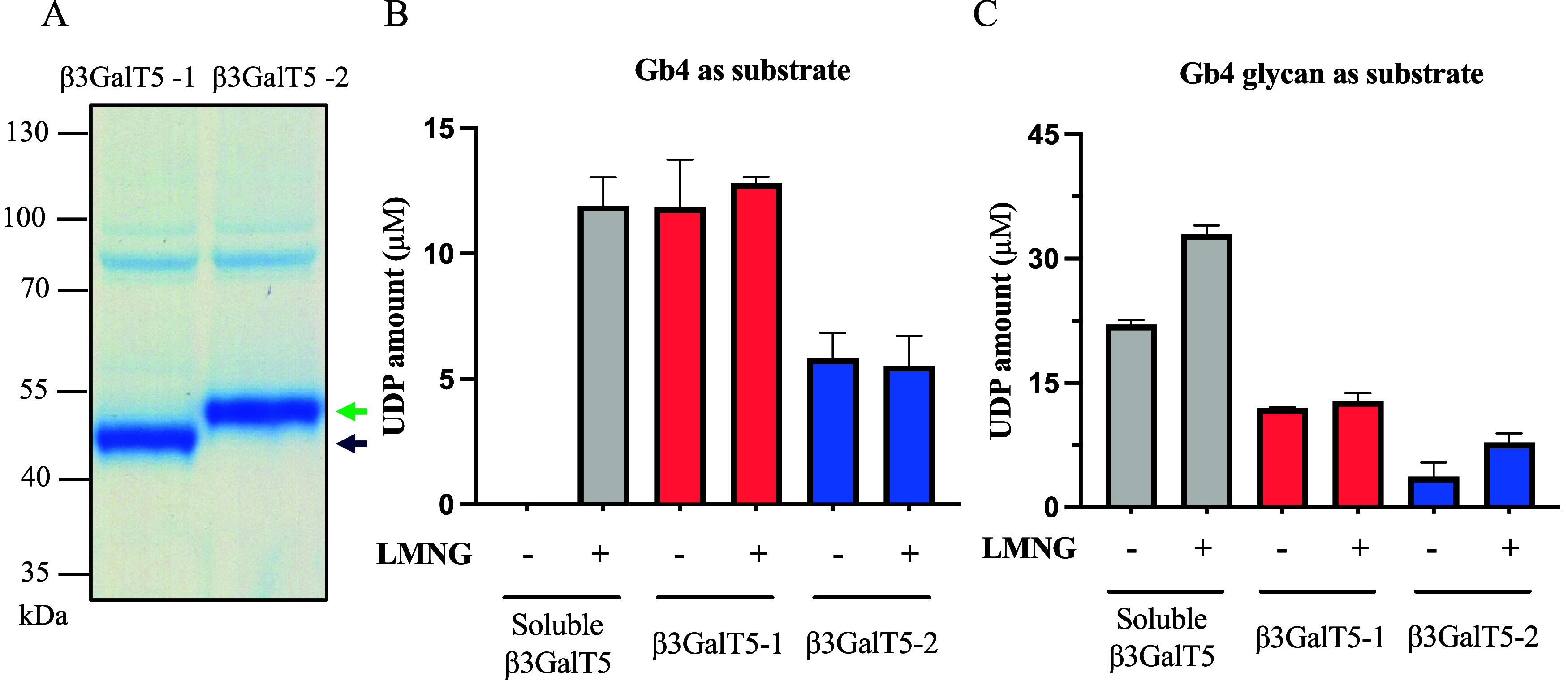
Purification and activity of β3GalT5 isozymes from HEK293 cells. A. Two membrane-bound β3GalT5 isozymes expressed in HEK293 cells were extracted by LMNG and further purified by anti-Flag beads. The blue and green arrows indicated β3GalT5–1 and β3GalT5–2, respectively. B, C. Enzymatic activities of membrane-bound β3GalT5–1 and β3GalT5–2, and the common soluble domain of both isozymes (soluble β3GalT5, residues N29–V310 of β3GalT5–1) using Gb4 and Gb4 glycan as substrates in the presence or absence of 1% LMNG. The results are presented as the mean ± standard deviation (SD) of three biological replicates.

To evaluate the difference in activity of the two isozymes involved in the synthesis of Gb5, two isozymes with a C-terminal Fag tag were expressed in HEK293 cells. To reduce the complexity during purification, organelles, including the Golgi apparatus where β3GalT5 localized, were specifically extracted by detergents and purified. The cell membrane and organelle fraction were separated by digitonin,^33^ and the organelle fractions were further screened with several detergents to optimize the extraction efficiency and the activity of the two isozymes (Figure S3). Of the 20 detergents screened, CHAPS (3-((3-cholamidopropyl) dimethylammonio)-1-propanesulfonate) and LMNG (lauryl maltose neopentyl glycol) showed better results, and LMNG showed higher extraction efficiency (Figure 2A). We also expressed the soluble domain of β3GalT5–1 (residues N29–V310, same sequence in β3GalT5–2) in HEK293 cells and found that it exhibited higher activity compared to membrane-bound β3GalT5–1 or β3GalT5–2 using the glycan of Gb4 as a substrate (Figure 2C). However, when the soluble β3GalT5–1 was incubated with Gb4 glycolipid and UDP-galactose in the absence of LMNG, no activity was observed, and an addition of a small amount of LMNG (1%) was required to have the enzymatic activity, perhaps due to the presence of LMNG to facilitate the orientation of Gb4 glycolipid as an acceptor for β3GalT5. Interestingly, β3GalT5–1 showed a 2-fold increase in activity compared to β3GalT5–2, regardless of whether Gb4 or Gb4 glycan was used as a substrate (Figure 2B and C). Based on these results, we decided to develop soluble β3GalT5–1 as a catalyst for the synthesis of SSEA-3 glycan.

### Specificity of Soluble β3GalT5–1 for Acceptor and Donor Substrates

Knocking down β3GalT5–1 in MCF-7 cells caused a major effect on the biosynthesis of globo-series glycans and *O*-glycans and a minor effect on *N*-glycans. To further explore the substrate specificity of β3GalT5–1, we investigated the activity of the soluble domain of β3GalT5–1 expressed in HEK293 toward disaccharides with different linkages and various types of sugars at the nonreducing end. It was observed that the disaccharides with terminal galactose (Gal), especially in β-1,3 linkage, were excellent substrates and those with terminal N-acetylglucosamine (GlcNAc) or N-acetylgalactosamine (GalNAc) were generally good substrates (Table 1). The glycan parts of both Gb4 and Lc3 were previously reported as native substrates for soluble β3GalT5.^34^ However, the enzyme toward the natural substrate Gb4 exhibited a low activity, which may be attributed to the presence of the ceramide tail that affects the orientation and accessibility of the glycan to β3GalT5–1 under the assay condition. This problem was mitigated with the addition of detergent LMNG to improve the accessibility of Gb4 glycan to β3GalT5 (Table 1). In the case of *O*-linked core structures, both type 3 and type 4 core structures were reported as substrates for human β3GalT5.^35−37^ To investigate the galactosylation preference on core 4 structures, we conducted enzymatic reactions using varying ratios of UDP-Gal to core 4 as the substrate, followed by analysis viaLC-MS/MS. The results show that β3GalT5 prefers the β1,3-arm of the *O*-glycan on the core 4 structure, achieving a conversion rate of 65.4% when the molar ratio of core 4 to UDP-Gal is 1:1. However, as the amount of UDP-Gal increases (UDP-Gal: core 4 = 2:1 and 4:1), galactosylation of both the β1,3-and β1,6-arms occurred, with conversion rates of 33.52 and 61.22%, respectively, compared to 62.72 and 38.12% when only a single Gal is added to the β1,3-arm. With an excess of UDP-Gal (UDP-Gal: core 4 = 8:1 and 20:1), more than 90% of the products showed galactosylation on both the β1,3-arm and β1,6-arm (Figure S4B and Table S2). In addition, the type 1 LacNAc glycan GlcNAc-β1-3-Gal-β1-3-GlcNAc was also a good acceptor substrate. Regarding donor specificity, soluble β3GalT5–1 exhibited a preference for UDP-Gal compared to other UDP-sugars using Gb4 glycan as an acceptor (Table 2). We also observed that UDP-Gal-6-aldehyde and UDP-Gal-6-azide were accepted at rates of 78 and 18%, respectively, compared to UDP-Gal (Table 2). The LC-MS results corroborated these findings, showing similar relative activity levels.

**Table 1:**
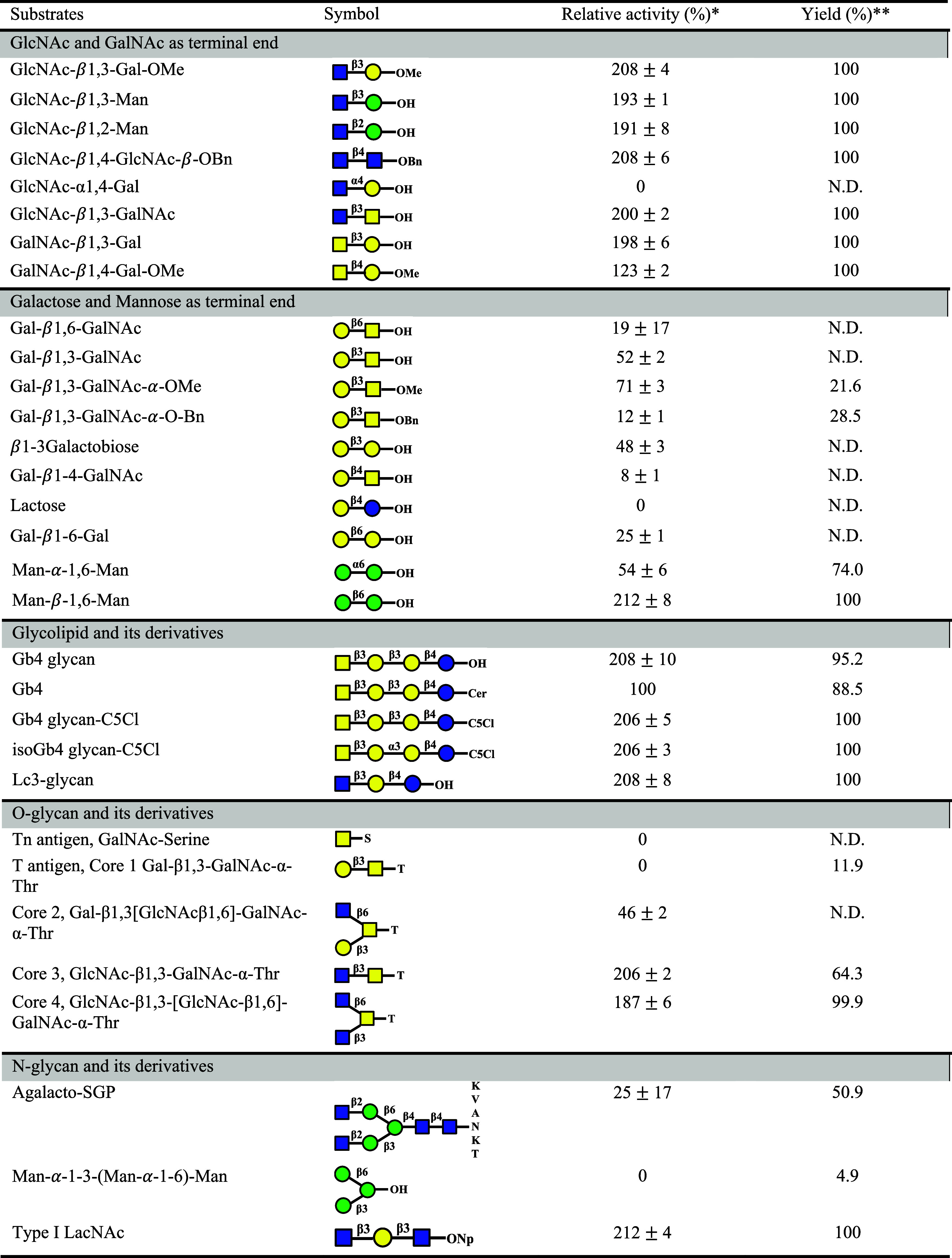
Substrate Specificity of Soluble β3GalT5-1 toward Disaccharides, Glycopeptides, and Glycolipids

a * Based on natural substrate Gb4 as a reference, all reactions were performed in 20 mM HEPES (pH 7.4) buffer containing 0.1 mM MnCl_2_, 0.1 mM UDP-Gal, 5 mM substrate, and 5 μM soluble β3GalT5–1 expressed from HEK293 cells. isoGb4 glycan: GalNAc-β1,3-Gal-α-1,3-Gal-β1–4-Glc, Lc3 glycan: GlcNAc-β1,3-Gal-β1,4-Glc, and type 1 LacNAc: GlcNAc-β1,3-Gal-β1,3-GlcNAc-*O*-nitrophenol. For the carbohydrate symbol, yellow circle: galactose (Gal), blue circle: glucose (Glc), green circle: mannose (Man), yellow square: GalNAc, and blue square: GlcNAc. The linker symbol stand for S: serine, T: threonine, K: lysine, V: valine, A: alanine, N: asparagine, Me: methyl group, Bn: benzyl group, and Np: nitrophenyl group. The results are presented as the mean ± SD of three biological replicates. ** All reactions were carried out using 10 μM acceptor substrate, 0.1 mM UDP-Gal, and 0.5 μM soluble β3GalT5–1, incubated at 37 °C for 24 h. The resulting mixture was then diluted 10-fold for LC-MS analysis. N.D.: not determined.

**Table 2:**
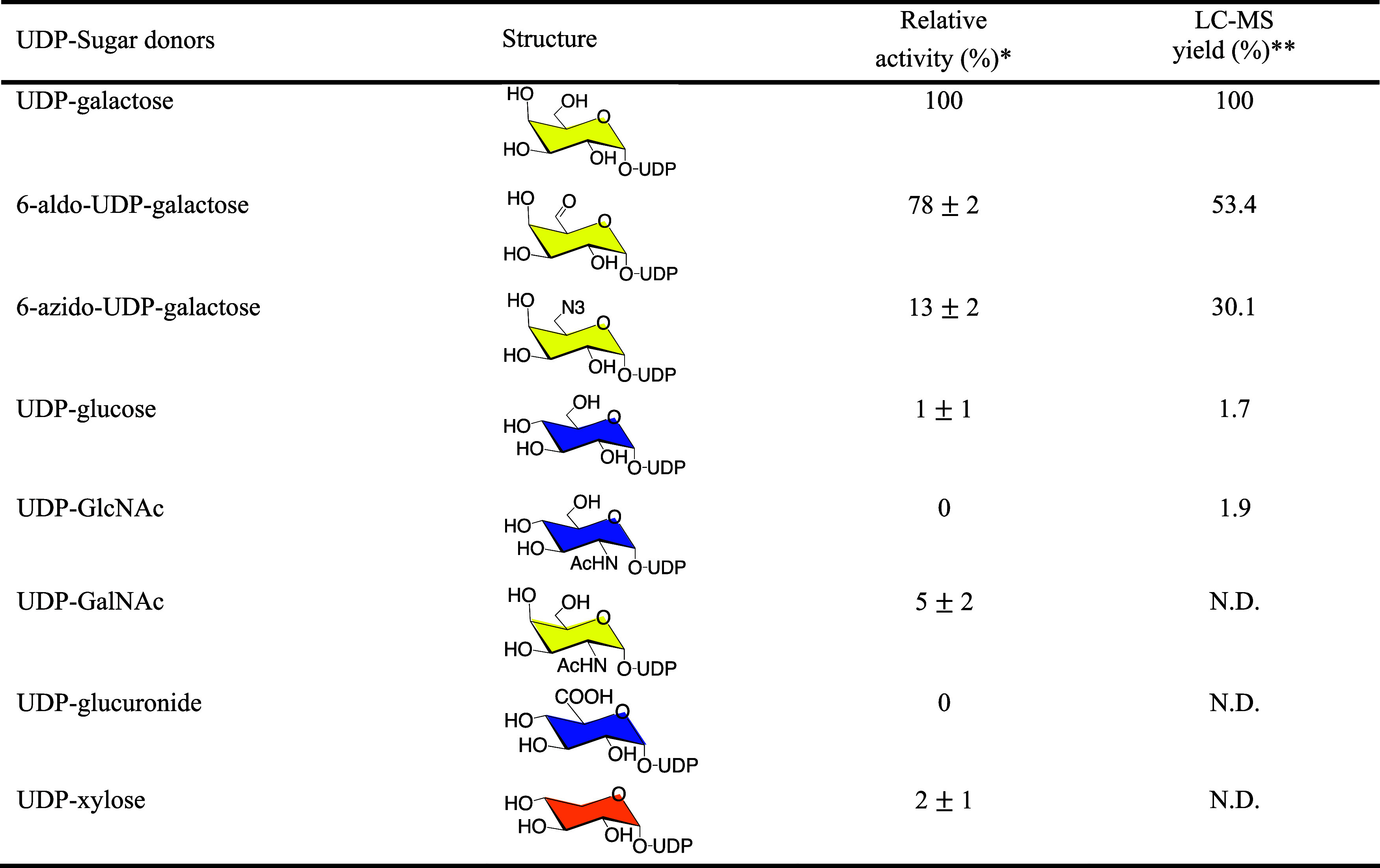
Donor Specificity of Soluble β3GalT5-1 toward UDP-sugars and UDP-Galactose Analogs

a * Based on UDP-Gal as a reference, all reactions were performed in 20 mM HEPES (pH 7.4) containing 0.1 mM MnCl_2_, 0.1 mM UDP-sugar or UDP-Gal analogs, 5 mM Gb4 glycan, and 5 μM soluble β3GalT5–1 expressed from HEK293 cells. The results are presented as the mean ± SD of three biological replicates. ** All reactions were carried out using 10 μM Gb4 glycan as the acceptor substrate, incubated with 0.1 mM UDP-sugar or UDP-Gal analogs, and 0.5 μM soluble β3GalT5–1 at 37 °C for 24 h. The resulting mixture was then diluted 10-fold for LC-MS analysis. N.D.: not determined.

### Human β3GalT5 Expressed in HEK293 or Insect Cells Is More Efficient than the Microbial LgtD in Catalyzing the Synthesis of SSEA-3 Glycan

In the chemo-enzymatic synthesis of globo-series glycans, the bifunctional enzyme LgtD from *Haemophilus influenzae* catalyzed the conversion of Gb4 to Gb5 glycan through β1,3-galactosylation.^38−40^ This galactosylation reaction was relatively slow, and the yield of Gb5 glycan with sugar nucleotide regeneration ranged from 63 to 74%, which was less efficient when compared to the other enzymatic steps in the large-scale synthesis of globo-series glycans.^25,41,42^ Based on the specificity study of β3GalT5, we next tried to identify an effective expression system to prepare the soluble domain of human β3GalT5–1 for the synthesis of SSEA-3 glycan. We initially expressed the enzyme in *Escherichia coli*. However, the enzyme was misfolded and could not be efficiently purified using the affinity tag (Figure S6A). We then coexpressed the soluble β3GalT5–1 with various molecular chaperones to promote folding in *E. coli*.^43,44^ Unfortunately, the yield was exceedingly low in all attempts except with the trigger factor (Figure S6B), but it did not have the proper enzymatic activity, probably due to the absence of post-translational glycosylation. We then expressed a fusion protein of β3GalT5–1 with the trigger factor in *E. coli* to facilitate the disulfide bond formation (Figure S6C). This approach yielded a substantial increase in the production of active β3GalT5–1, reaching 20–25 mg/L with activity comparable to that of LgtD (Table S3). Upon cleavage of the trigger factor using thrombin, the activity of β3GalT5–1 was doubled as compared to LgtD but remained ten times less efficient than human β3GalT5–1 expressed in insect cells (Figure S6D). The soluble domain of β3GalT5–1 expressed in HEK293 cells contains complex-type glycoforms with three *N*-glycosites. To assess the effect of glycosylation on the enzymatic activity, the soluble domain of β3GalT5–1 was also expressed in insect cells (Sf9 cells) to produce the enzyme with mainly paucimannose glycoforms and in the presence of kifunensine to generate the high mannose-type glycoforms, which were further treated with Endo-H to obtain mono-GlcNAc β3GalT5–1. The deglycosylated β3GalT5–1 was also prepared after PNGase F digestion; however, the activity was significantly reduced to less than 50% activity compared to the untreated, a result similar to that from *E. coli*, while the high mannose (Man) and the mono-GlcNAc glycoforms had 2-fold higher activity than the paucimannose structure (Figure S6E). We also expressed the soluble form of β3GalT5–1 in yeast (*Pichia pastoris*); however, the purified proteins had no function, probably due to improper glycosylation and misfolding. To compare the catalytic activity of LgtD and human β3GalT5–1 in converting Gb4 to SSEA-3 glycan, the kinetic parameters of soluble β3GalT5–1 expressed in *E. coli*, HEK293, and insect cells (Sf9 cells) were determined by monitoring the amount of UDP released from UDP-Gal. When allyl-Gb4 glycan was used as the substrate, the *K*_m_ and *k*_cat_ values for human β3GalT5–1 from HEK293 cells were 0.64 ± 0.3 and 3.3 ± 0.1 per minute, respectively, compared to 265.4 ± 88.3 and 0.046 ± 0.003 per minute for LgtD (Table 3). The turnover rate of human β3GalT5–1 was approximately 30,000-fold higher than that of LgtD.

**Table 3:** Kinetic Parameters for Human Soluble β3GalT5-1 and LgtD

enzyme^a^	expression host	substrate	*K*_m_ (mM)	*k*_cat_ (min^–1^)	*k*_cat_/*K*_m_ (min^–1^ mM^–1^)
LgtD	*E. coli*	Allyl-Gb4 glycan	265.4 ± 88.3	0.046 ± 0.003	1.8 × 10^–4^ ± 3.4 × 10^–5^
soluble β3GalT5–1	Insect		0.66 ± 0.4	3.5 ± 0.4	5.3 ± 1.0
	HEK293		0.64 ± 0.3	3.3 ± 0.1	5.2 ± 3.0

a The enzymatic reaction of LgtD was performed in MOPS (pH 7.0) buffer with 1 mM MgCl_2_, 0.1 mM UDP-Gal, and a 2-fold serial concentration of allyl-Gb4 (0–500 mM). The soluble β3GalT5–1 reaction was performed in HEPES buffer with 0.1 mM MnCl_2_, 0.1 mM UDP-Gal, and a 2-fold serial concentration of allyl-Gb4 (0–4 mM). The results were fitted to the Michaelis–Menten enzyme kinetics model using GraphPad Prism10 and are presented as the mean ± SD of three biological replicates.

### Optimization of Soluble β3GalT5–1 Expressed in Insect Cells for the Chemo-Enzymatic Synthesis of SSEA-3 Glycan

To efficiently synthesize the globo-series glycans using soluble β3GalT5–1, several problems must be resolved, and the most important one is to ensure proper glycosylation of the enzyme to retain the activity. While the expression yield of the enzyme from insect cells was approximately 8–10 mg/L, the activity of soluble β3GalT5–1 expression from insect cells was similar to that from HEK293 cells for the conversion of Gb4 to SSEA-3 glycan, as confirmed by ultra-performance liquid chromatography (UPLC). However, when we replaced LgtD with soluble β3GalT5–1 from insect cells and employed a sugar nucleotide regeneration method to synthesize the SSEA-3 glycan, a byproduct with additional galactose on the SSEA-3 glycan was observed, as confirmed by thin-layer chromatography (TLC) and MS. To address this issue, we optimized the amount of soluble β3GalT5–1 and the molar ratio of Gb4 glycan to UDP-Gal under various conditions. We found that when the molar ratio of UDP-Gal to Gb4 glycan was adjusted to 0.8 and 1, no side product was observed in TLC and MS analyses within 45 h. However, with an excess amount of soluble β3GalT5–1 and longer reaction time, side product formation increased after 69 h (Table S4). Therefore, we thought that the molar ratio of Gb4 glycan to soluble β3GalT5–1 should range from 8000 to 10,000, while the ratio of Gal (which then converted to UDP-Gal through sugar nucleotide regeneration) to Gb4 glycan should range from 0.9 to 1. With this condition, we can minimize the side product formation, and the enzymatic reaction can be completed within a day.

### Structure of Soluble β3GalT5–1 Expressed in Insect Cells

**Figure 3:**
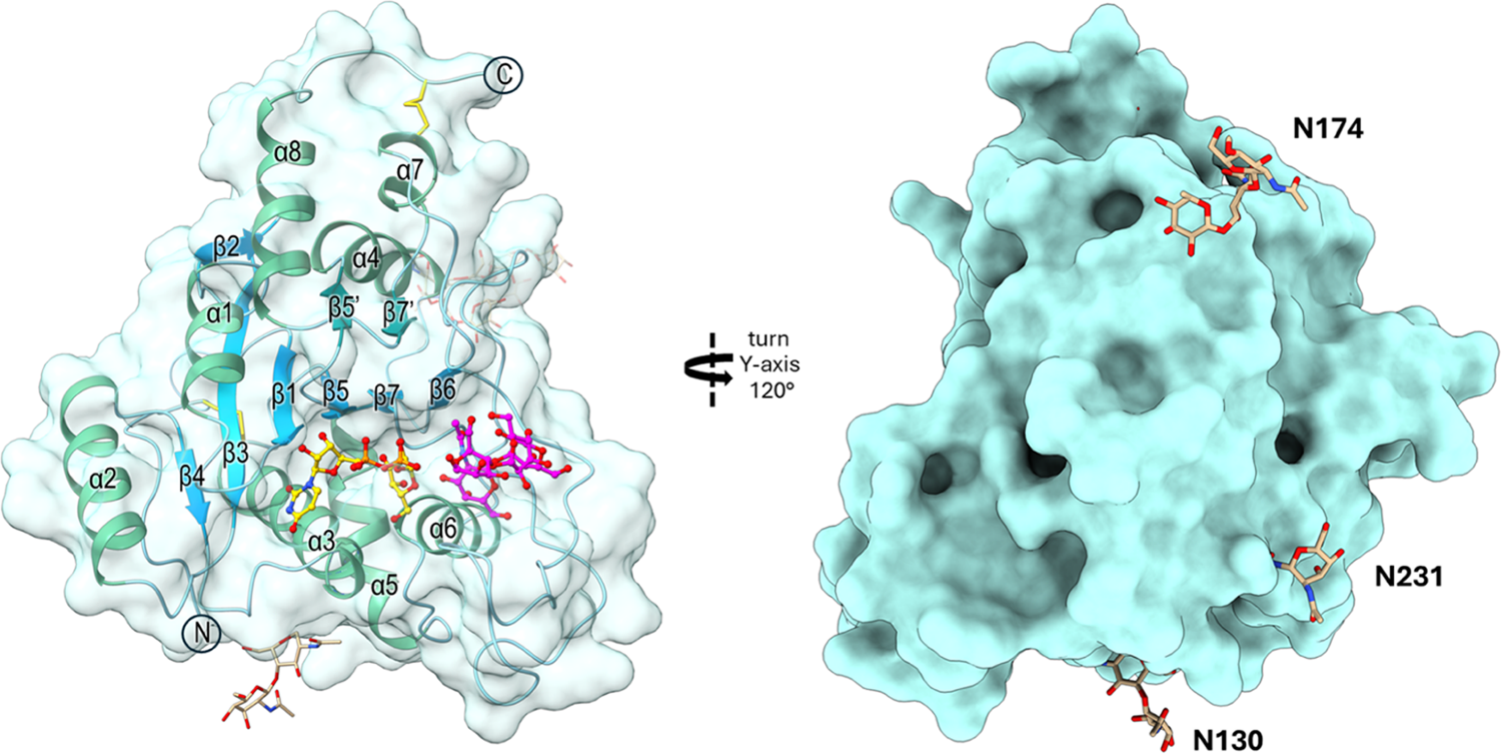
Surface representation of soluble β3GalT5–1 with overall structure and *N*-glycosylation. Surface representation of the enzyme β3GalT5–1, highlighting its substrate binding cleft and *N*-glycosylation sites. The protein surface is shown in pale turquoise, while the α-helix is shown in mint, the β-sheet is in dark cyan, and the two disulfide bonds (Cys52-Cys146 and Cys276-Cys307) are in yellow. The divalent ion manganese appears as a purple sphere. The donor substrate UDP-Gal is represented by gold sticks, and the acceptor substrate Gb4 glycan is represented by pink sticks. The three glycosites (N130, N174, and N231) are highlighted with brown sticks. The electron density map reveals the following glycosylation patterns: site N130 up to two GlcNAc residues, site N231 up to one GlcNAc residue, and site N174 displays paucimannose glycosylation.

The X-ray structure of soluble β3GalT5–1 from insect cells cocrystallized with UDP-Gal was determined through sulfur single-wavelength anomalous dispersion phasing, with a resolution of 2.20 Å. In addition, the structures of soluble β3GalT5–1 in complex with either substrates or products through molecular replacement, employing the sulfur derivative structure as a template, were also determined. The soluble enzyme is composed of residues Phe31-Pro308, although only residues Asp41-Pro308 are clearly visible in the electron density maps. The catalytic domain displayed a mixed α/β Rossmann-like fold, typical of the GT-A glycosyltransferases family, including a central seven-stranded β-sheet (β1−β7) encircled primarily by α-helices (α1−α7), a two-stranded antiparallel β-sheet (β5′ and β7′), and an additional 13-residue α-helix (α8) at the C-terminal. Two disulfide bonds, Cys52-Cys146 and Cys276-Cys307, along with the glycans at all three glycosylation sites (Asn130, Asn174, and Asn231) were identified. Both the donor and acceptor sugar molecules were positioned near the center of the binding cleft (Figure 3). As expected, based on the analysis of *N*-glycans with mass spectrometry, the soluble domain of β3GalT5–1 expressed from insect cells contains mainly paucimannose glycoforms, whereas the glycoforms from HEK293 cells are mainly complex type (Figure S7).

### Identification of S66A β3GalT5–1 with Enhanced Catalytic Efficiency through Site-Specific Alanine Scan for the Synthesis of Gb5 Glycan from Gb4 Glycan

To improve the catalytic efficiency of soluble β3GalT5–1 in chemo-enzymatic synthesis, we introduced site-directed mutagenesis of residues that interact with UDP-Gal and Gb4 glycan, followed by the evaluation of their activity through an enzymatic assay. Remarkably, the activities of T64A, S66A, and Q69A variants of β3GalT5–1, which interacted with UDP-Gal, were significantly increased. Particularly intriguing were the findings that S66A and Q69A exhibited approximately 3-fold and 1.5-fold increases in activity compared to the wild-type enzyme, whereas the activity of other mutants decreased significantly (Figure S8). Furthermore, we conducted mutations of the S66 and Q69 positions with alternative amino acids. When the S66 position was substituted with valine, threonine, and glycine, a slight reduction in activity was observed compared to alanine but remained higher than that of the wild type. When the Q69 position was replaced with methionine, its activity matched that of alanine and exhibited an approximately 2-fold increase in activity compared to the wild type. In comparing the kinetic parameters of these single mutants (S66A, Q69A, and Q69M) and double mutants (S66A/Q69A or S66A/Q69M) using UDP-Galas donor and allyl-Gb4 glycan as acceptor, we observed a significant reduction in the enzymatic activities of β3GalT5–1 with S66A/Q69A or S66A/Q69M double mutations compared to the wild type. Among the single mutants, S66A displayed the highest efficiency, with *K*_m_ values for UDP-Gal and allyl-Gb4 glycan of 0.35 and 1.20 mM, *k*_cat_ values at 16.55 and 4.66 per minute, and *k*_cat_/*K*_m_ values at 46.86 and 3.89 min^–1^ mM^–1^, respectively. Notably, S66A exhibited a decreased binding affinity for UDP-Gal and Gb4 glycan, but it showed an increased turnover rate and efficiency for Gb4 glycan when compared to the wild-type β3GalT5–1. The specificity for UDP-Gal remained unchanged compared to that of wild type (Table 4).

**Table 4:** Kinetic Parameters of Soluble β3GalT5-1 Mutants

β3GalT5–1^a^	substrate	*K*_m_ (mM)	*k*_cat_ (min^–1^)	*k*_cat_/*K*_m_ (min^–1^ mM^–1^)
WT	Allyl-Gb4	1.40 ± 0.2	2.69 ± 0.2	1.92 ± 1.0
	UDP-Gal	0.14 ± 0.04	7.51 ± 0.7	52.18 ± 17.6
S66A	Allyl-Gb4	1.20 ± 0.2	4.66 ± 0.3	3.89 ± 1.9
	UDP-Gal	0.35 ± 0.1	16.55 ± 2.5	46.86 ± 24.5
Q69A	Allyl-Gb4	1.21 ± 0.3	0.72 ± 0.07	0.60 ± 0.2
	UDP-Gal	0.30 ± 0.05	2.98 ± 0.2	9.88 ± 3.8
Q69M	Allyl-Gb4	0.42 ± 0.3	0.64 ± 0.1	1.52 ± 0.5
	UDP-Gal	0.21 ± 0.05	3.51 ± 0.3	16.93 ± 6.2
S66A/Q69A	Allyl-Gb4	1.11 ± 0.3	0.47 ± 0.05	0.42 ± 0.2
	UDP-Gal	0.32 ± 0.09	1.64 ± 0.2	9.35 ± 2.7
S66A/Q69M	Allyl-Gb4	1.65 ± 0.4	1.49 ± 0.1	0.90 ± 0.4
	UDP-Gal	0.27 ± 0.06	4.64 ± 0.4	17.05 ± 6.8

a All reactions were conducted in a buffer containing 20 mM HEPES (pH 7.4) and 0.1 mM MnCl_2_, with 0.5 μM soluble β3GalT5–1 mutants expressed in HEK293 cells. To measure the kinetic parameters of ally-Gb4 or UDP-Gal, reactions were set up with a fixed concentration of 10 mM UDP-sugar or 2 mM Gb4 glycan and a 2-fold serial dilution of allyl-Gb4 glycan (0–4 mM) or UDP-Gal (0–1 mM). The resulting data were analyzed using GraphPad Prism10 to fit the Michaelis–Menten enzyme kinetics model. The results are presented as the mean ± SD of three biological replicates.

**Figure 4:**
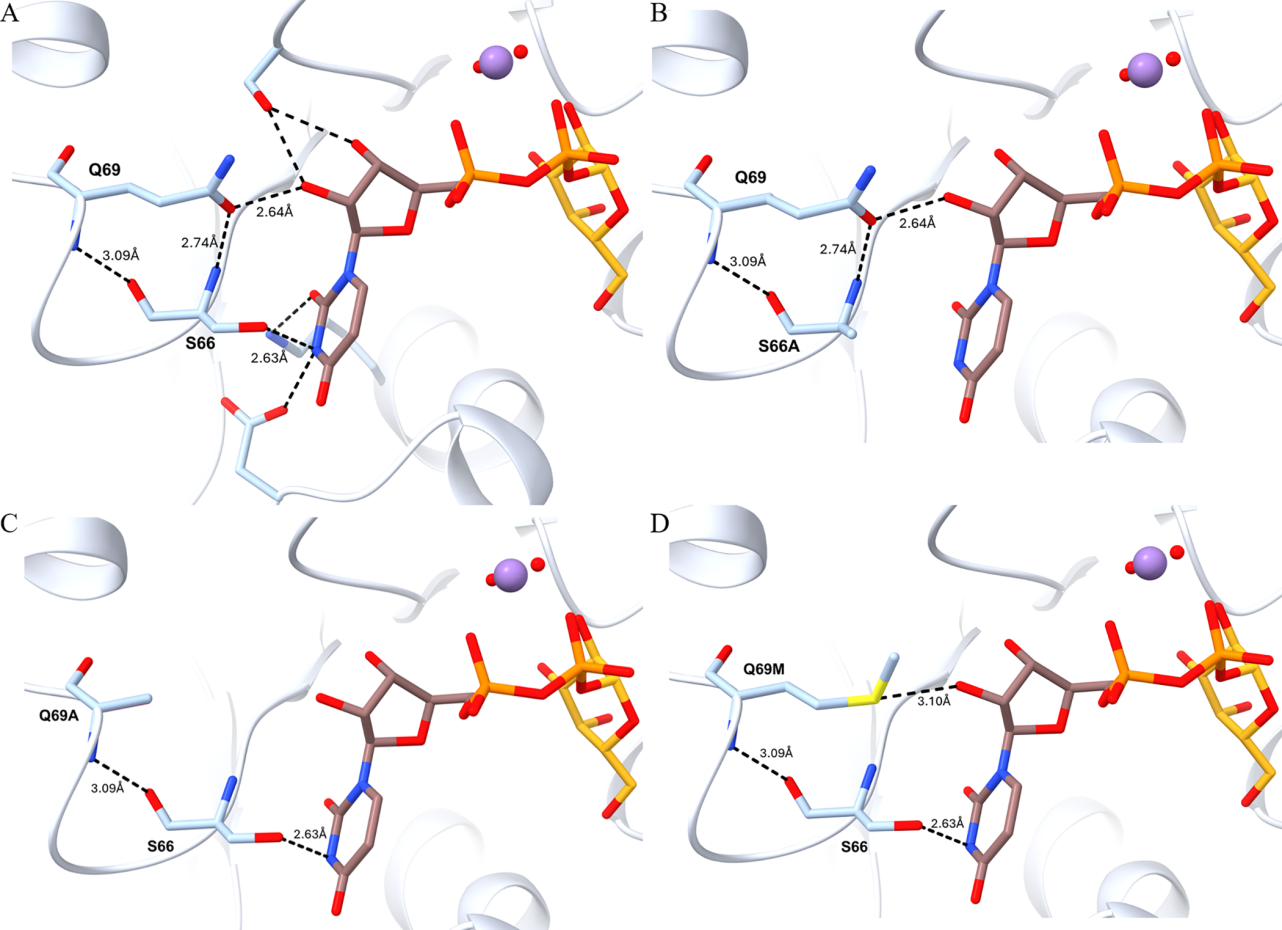
Mutations of S66A and Q69A/M in soluble β3GalT5–1 affect interactions with UDP. A. Residues S66 and Q69 in wild-type β3GalT5–1 interact (dashed lines) with the UDP moiety only. Side chains of residues involved are shown as sticks. UDP is shown as brown sticks, Gal as gold sticks, divalent ion Mn^2+^ as a purple sphere, and water molecules as red spheres. B. Modeling structure of S66A mutant. C. Modeling structure of Q69A mutant. D. Modeling structure of Q69M. All modeled mutant structures are based on the wild-type β3GalT5–1 structure.

To understand the higher activity of the S66A mutant, we performed molecular modeling of the wild-type enzyme in complex with UDP-Gal. It was shown that the S66 backbone carbonyl group interacted with the backbone NH of Q69 through hydrogen bonding. This interaction remains the same even with S66A and/or Q69A/M mutations (Figure 4A). In the S66A mutant, the interaction between 66A and the N3 of uracil in the UDP moiety was abolished (Figure 4B). In addition, the hydrogen bonding between the S66 carbonyl group and the Q69 NH group no longer existed in the Q69A and Q69M mutations (Figure 4C and D). The lack of S66A or Q69A/M side chain interactions with UDP led to decreased binding to UDP-Gal. Consequently, wild-type β3GalT5 binds to UDP-Gal more strongly (*K*_m_ = 0.14 mM) compared to the S66A (*K*_m_ = 0.35 mM) and Q69A (*K*_m_ = 0.30 mM) mutants. Moreover, a similar *K*_m_ value for UDP-Gal was observed for the Q69M, S66A, and Q69A mutants. Although structural analysis can only directly explain the *K*_m_ value of the kinetic parameters, the loss of the S66A side chain interaction with the UDP moiety might contribute to the higher turnover rate observed for the S66A mutant (*k*_cat_ = 16.55 UDP-Gal/min; Table 4). The weaker binding to UDP potentially contributes to a faster release of UDP and faster reaction as more UDP-Gal is accepted by the enzyme in each catalytic cycle, and the UDP generated is more quickly released from the enzyme. In enzymatic synthesis, the concentrations of substrates usually are higher than their *K*_m_ values; consequently, the enzyme is saturated by substrates in the reaction, and the efficiency is thus determined by the *k*_cat_ value (the turnover rate). This enhanced catalytic efficiency means that the S66A mutants reduced the time needed to synthesize the SSEA-3 glycan.

The substrate specificity of soluble S66A β3GalT5–1 closely resembled that of wild-type soluble β3GalT5–1 (Figure S5). Notably, the relative activity for disaccharides with Gal at the terminal end was elevated compared to the wild type. This increase in activity could lead to an additional galactosylation in the synthesis of Gb4 glycan. However, this side reaction can be minimized by adjusting the molar ratio of Gal to Gb4 glycan ranging from 0.8 to 1. This ratio is consistent with the conditions used for synthesizing allyl-Gb5 via sugar nucleotide regeneration with the wild-type soluble β3GalT5–1. Moreover, by comparing the thermostability of wild type and S66A mutant of β3GalT5–1, based on the melting temperature (*T*_m_) calculated by Boltzmann and derivative methods, the WT β3GalT5 expressed from insect cells showed *T*_m,Boltzmann_: 42.95 °C and *T*_m,Derivative_: 45.15 °C, whereas the S66A mutant showed *T*_m,Boltzmann_: 42.42 °C and *T*_m,Derivative_: 44.72 °C (Figure S9). The melting temperature between the WT and S66A mutant showed similar thermostability.

### Enzymatic Synthesis of Ally-Gb5 Glycan from Allyl-Gb4 Glycan with Soluble β3GalT5–1-S66A

**Figure 5:**
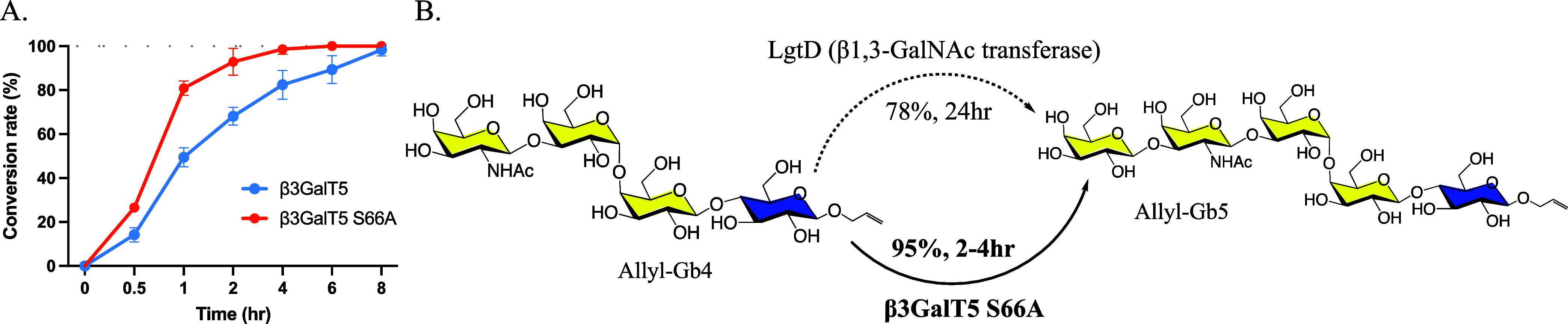
Optimized procedure for the enzymatic synthesis of SSEA-3 glycan. A. β3GalT5–1 and β3GalT5–1-S66A catalyzed the synthesis of 2-AB-Gb5 glycan at various time points. The results are presented as the mean ± SD of three biological replicates. B. Synthesis of allyl-SSEA-3 glycan using β3GalT5–1-S66A and LgtD.

To verify the relative activity and kinetic parameters of β3GalT5–1-S66A, we performed the chemo-enzymatic synthesis of allyl-Gb5 glycan using the S66A mutant expressed from insect cells as a catalyst and 2-aminobenzamide (2-AB) labeled Gb4 glycan as a substrate, and the reaction was monitored by UPLC. Within 2 h, approximately 90% of Gb4 glycan was converted to SSEA-3 glycan, whereas the wild-type β3GalT5–1 achieved only a 60% conversion. In 4 h, almost all Gb4 glycan was converted to SSEA-3 glycan by β3GalT5–1-S66A, while the reaction time required for wild-type β3GalT5–1 was doubled (Figure 5A). In a preparative-scale synthesis starting with 100 mg of allyl-Gb4 glycan and β3GalT5–1-S66A along with other enzymes and reagents, nearly all of the allyl-Gb4 glycan was transformed into allyl-SSEA-3 glycan within 2 h as monitored by TLC. The yield of allyl- SSEA-3 glycan after purification through a C-18 column and confirmed with MS and NMR was 95% (Figure 5B). With the improved conversion efficiency from allyl-Gb4 to allyl- SSEA-3 glycan, this method should enable the large-scale one-pot synthesis of SSEA-4 and Globo-H glycans, as demonstrated previously, with lower cost and less reaction time.

### Enzymatic Synthesis of Ally-Globo-H Glycan from Allyl-Lactose Using Soluble β3GalT5–1-S66A Coupled with Sugar Nucleotide Regeneration

**Figure 6:**
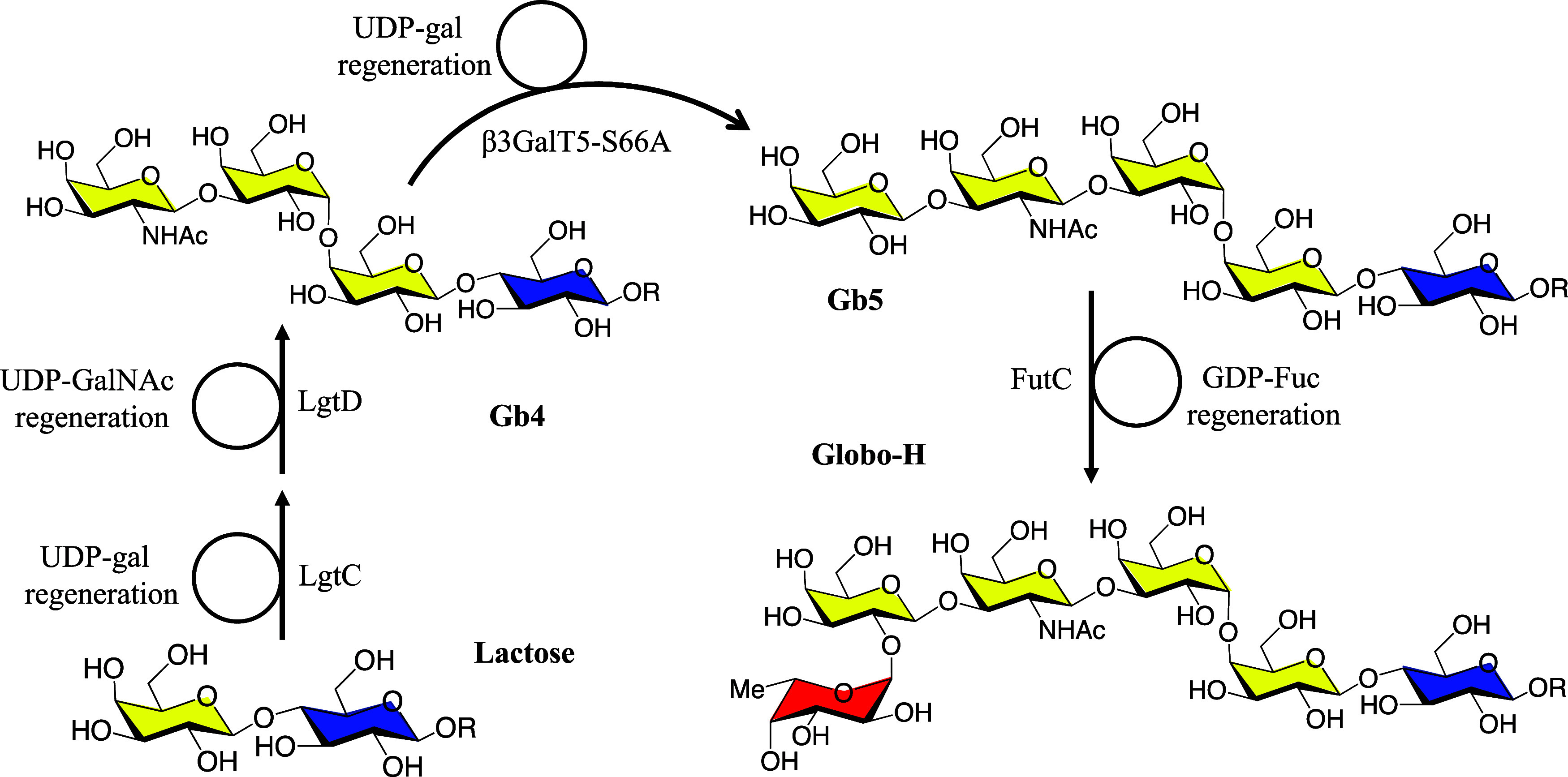
Enzymatic synthesis of allyl-Globo-H glycan from allyl-lactose with sugar nucleotide regeneration. Gb4 glycan was synthesized through the sequential action of α-1,4-galactosyltransferase (LgtC) and β-1,4-*N*-acetylgalactosaminyltransferase (LgtD) by transferring UDP-Gal to lactose and UDP-GalNAc to Gb3, achieving a synthetic yield of 94%. Subsequently, Gb5 glycan was synthesized using β-1,3-galactosyltransferase (soluble β3GalT5–1 with S66A mutation) with a synthetic yield of 95%. Finally, Globo-H glycan was synthesized by using α-1,2-fucosyltransferase (FutC), resulting in a synthetic yield of 95%.

To synthesize the allyl-glycan of Globo-H, the starting material allyl-lactose was mixed with other components following the procedure described previously,^17^ except the galactosyltransferase used for converting allyl-Gb4 to allyl-Gb5 glycan. In brief, 500 mg of allyl-lactose in a final volume of 22.3 mL solution containing 25 mM Tris-HCl (pH 7.5), 15 mM magnesium chloride (MgCl_2_), 50 mM Gal, 5 mM adenosine triphosphate (ATP), 5 mM uridine 5′-triphosphate (UTP), and 100 mM phosphoenol-pyruvate (PEP) was mixed with all required enzymes, including 0.2 mM UDP-sugar pyrophosphorylase (AtUSP), 0.4 mM galactose kinase (GalK), 0.1 mM pyruvate kinase (pykF), 1 mM pyrophosphatase (PPA), and 1 mM α-1,4-galactosyltransferase (LgtC). The reaction was incubated at 30 °C with gentle shaking and monitored with thin-layer chromatography using the solvent system butanol/acetic acid/water (5:3:2 v/v/v) until conversion to ally-Gb3 glycan was completed. For the conversion of allyl-Gb3 to allyl-Gb4 glycan, the solution was further added to the following reagents and enzymes: 15 mM Tris-HCl (pH 7.5), 50 mM GalNAc, 15 mM MgCl_2_, 5 mM ATP, 5 mM UTP, 100 mM PEP, 0.1 mM pykF, 1 mM PPA, 0.2 mM *N*-acetylhexosamine 1-kinase (NahK), 0.4 mM *N*-acetylglucosamine-1-phosphate uridyltransferase (GlmU), and 0.2 mM LgtD until the reaction was finished, and then the following reagents and enzymes were added to the mixture to generate ally-Gb5 (SSEA-3) glycan: 25 mM Tris-HCl (pH 7.5), 10 mM MgCl_2_, 10 mM manganese(II) chloride (MnCl_2_), 50 mM Gal, 5 mM ATP, 5 mM UTP, 100 mM PEP, 0.2 mM GalK, 0.2 mM AtUSP, 0.1 mM pykF, 0.5 mM PPA, and 0.06 mM S66A β3GalT5–1. When allyl-Gb4 glycan was fully converted to allyl-SSEA-3 glycan, the mixture was then passed through 10K MWCO AmiconUltra, washed with water several times, and further purified by C-18 column with a gradient concentration of methanol. To synthesize allyl-Globo-H glycan, 500 mg of purified allyl-SSEA-3 glycan was dissolved in 10.2 mL of the solution containing 0.6 mM pykF, 1.5 mM PPA, 0.4 mM fucokinase/GDP-fucose pyrophosphorylase (FKP), 0.4 mM fucosyltransferase (FUTC), 55 mM fucose, 5 mM ATP, 5 mM GTP, 25 mM Tris-HCl (pH 7.5), 10 mM MgCl_2_, and 100 mM PEP. The product allyl-Globo-H glycan was then purified by a C-18 column with a gradient of methanol and further confirmed the structure by NMR and mass spectrometry. The overall yield of allyl-Globo-H glycan from allyl-lactose is 85% (Figure 6).

## Conclusions

Human galactosyltransferase β3GalT5 is the key enzyme that catalyzes the galactosylation of Gb4 to the cancer-associated SSEA-3 (Gb5), leading to the subsequent synthesis of the other two globo-series GSLs, SSEA-4 and Globo-H. The globo-series GSLs are often exclusively expressed on the surface of various types of cancer, and their glycans are considered targets for cancer therapies. The development of a practical enzymatic method for the synthesis of globo-series glycans is important, as it will provide sufficient materials for the evaluation of these glycans as targets for clinical development. Previously, we developed an enzymatic method for the large-scale synthesis of Globo-H glycan to enable the manufacture of a cancer vaccine for the phase 3 clinical trial. In this enzymatic process, the microbial galactosyltransferase LgtD was used in the synthesis of SSEA-3 glycan but was significantly less efficient than the human galactosyltransferase β3GalT5. However, human β3GalT5 is a glycoprotein with three *N*-glycosites and exists as two isozymes with only a difference in four N-terminal amino acids; it is not clear about the roles of each isozyme and glycosylation on the proteins in catalysis and synthesis of globo-series glycolipids. In this study, we developed a precise dsiRNA approach to specifically knockdown each of the two isozymes of human β3GalT5 in MCF-7 cells and identified β3GalT5–1 as the main isozyme responsible for the synthesis of SSEA-3 (Gb5) from Gb4 in cancer cells. We also found that among the various glycosylation patterns, the glycoforms expressed in human (complex type) and insect cells (Sf9) (paucimannose type) are more active than the glycoforms with other types of glycosylation patterns from other species including yeast (such as *Pichia pastoris*). We therefore successfully expressed the common soluble domains of β3GalT5–1 and β3GalT5–2 (N29–V310) in insect cells as the most active glycoforms and investigated the substrate specificity. Furthermore, we performed a site-specific alanine scan and mutation in the binding site and identified the S66A mutant of β3GalT5–1 with a 10-fold increase in efficiency compared to the microbial enzyme LgtD. Molecular modeling of wild-type β3GalT5–1 and its S66A mutant revealed that the loss of key interactions with UDP-Gal in the mutant decreases binding affinity for UDP-Gal (higher *K*_m_ value) but enhances turnover rate (higher *k*_cat_) with no effect on binding to the glycan acceptor. With the soluble domain of β3GalT5–1 readily available from insect cell expression, it can be used with other enzymes for the efficient synthesis of oligosaccharides and facilitate the evaluation of their translational applications.

## Supporting Information Available

Synthetic and experimental procedures, supporting figures and tables displaying characterization and structural analyses and crystallographic information are included (PDF)
