# Supplementary material for: Expression of Human β3GalT5–1 in Insect Cells as Active Glycoforms for the Efficient Synthesis of Cancer-Associated Globo-Series Glycans
Source: J Am Chem Soc. 2025 Mar 25;147(13):10864–74. doi: 10.1021/jacs.4c11723 (PMC11969553; doi:10.1021/jacs.4c11723)
Supplement: Supplementary file 1 — ja4c11723_si_001.pdf [file ja4c11723_si_001.pdf]

Supplementary Information

Expression of Human β3GalT5-1 in Insect Cells as Active Glycoforms for the Efficient Synthesis of Cancer-Associated Globo-Series Glycans

Chih-Chuan Kung[a, ‡], Jennifer M. Lo[a, ‡], Kuo-Shiang Liao[a], Chung-Yi Wu[a], Li-Chun Cheng[a], Cinya Chung[a], Tsui-Ling Hsu[a], Che Ma[a*] and Chi-Huey Wong[ab*]

[a]Genomics Research Center, Academia Sinica, Taipei 11529, Taiwan
[b]Department of Chemistry, The Scripps Research Institute, La Jolla, California 92037, United States

[‡]These authors contributed equally
*Email: wong@scripps.edu or cma@gate.sinica.edu.tw

Table of contents

Material and methods.. S2
Supplementary Figures and Tables..S8
Figure S1. Protein and RNA sequences of isozymes β3GalT5-1 and β3GalT5-2.......S8
Table S1. dsiRNA sequences designed to target β3GalT5-1......................................S9
Figure S2. Glycosylation changed after knocking down of β3GalT5-1 in breast cancer cells...S10
Figure S3. Detergent screening of β3GalT5-1 or β3GalT5-2 expressed from HEK293 cells...S13
Figure S4. Products confirmation by LC-MS..S14
Table S2. Galatosylation of O-linked core 4 structure through soluble β3GalT5..S21
Figure S5. NMR spectrum of Allyl-Gb5, Gb5-C5Cl, Gal-β1, 3-GlcNAc-β1, 3-Gal-OMe and Gal-β1, 3-Man-β1, 6-Man..S22
Figure S6. Expression of recombinant soluble β3GalT5-1 (N29-V310) fused with N-terminal molecular chaperone trigger factor (TF) in E. coli......................................S32
Table S3. Comparison of Activity Between TF-β3GalT5-1 (N29-V310) and LgtD.S33
Table S4. Optimized Synthesis of SSEA-3 Using Soluble β3GalT5-1.......................S33
Figure S7. Compositions of the three N-glycosites (N-130, N-174, N-231) in β3GalT5-1 expressed from insect and mammalian cells...S34
Figure S8. Alanine screening of the residues interacting with UDP-galactose.........S35
Table S5. Substrate specificity of soluble S66A β3GalT5-1....................................S36
Figure S9. Thermostability of soluble domain β3GalT5-1 WT and S66A mutant....S37

References..S38

Material and methods.

General Information

The allyl-globo series glycans and the UDP-Gal analogs were synthesized according to the reported procedures. [1] Others glycans were purchased from BIOSYNTH™. All cell lines were from ATCC except Expi293™ and ExpiSf9™ insect cells from Invitrogen. Luminance signal was recorded on BMG CLARIOstar™ Plus microplate reader. UPLC spectra were recorded on Waters ACQUITYUPLC H class spectrometer using ACQUITY UPLC Glycan BEH Amide 1.7 um, 2.1X150 mm column with gradient ACN: ammonium formate (pH4.4) and detection at Ex 310 nm; Em 420 nm. Flow cytometry spectra were recorded on BD FACSCanto™ Flow Cytometry. PCR amplicons were recorded on Applied Biosystems QuantStudio™ 5 Real-Time PCR System whereas droplet amplicons were recorded and analyzed on Biorad QX200™ Droplet Reader and the QuantaSoft Pro™ software.

Cell line

For the cultivation of the expression cell lines, Expi293 human embryonic kidney cells and ExpiSf9 insect cells were grown in Expi293 Expression Medium and ExpiSf ™ CD Medium, following the manufacturer's instructions. Human mammary epithelial cells, hTERT-HME1 (ATCC CRL-4010), were maintained in MEGM™ Mammary Epithelial Cell Growth Medium with the provided supplements (Lonza). MCF-10A (ATCC CRL-10317) cells were cultured in DMEM-F12 medium (Gibco) supplemented with 10% Hyclone defined fetal bovine serum (Cytiva), 100 μg/mL epidermal growth factor (EGF) (Gibco), 1 mg/mL hydrocortisone (Sigma), 10 mg/mL insulin (Gibco), 100 ng/mL cholera toxin (Sigma), and 100 U/mL penicillin G and 100 μg/mL streptomycin (PS) (Gibco). MCF-7 (ATCC HTB-22) cells were grown in RPMI 1640 (Gibco) with 10% FBS, 1% MEM Non-Essential Amino Acids (Gibco), and 1% Antibiotic-Antimycotic (Gibco). MDA-MB-231 (ATCC HTB-26) cells were cultured in DMEM with 10% FBS and 1% antibiotic-antimycotic. As for lung normal epithelial cells and cancer cells, NuLi-1 (ATCC CRL-4011) and BEAS-2B (ATCC CRL-3588) were nurtured in BEGM™ Bronchial Epithelial Cell Growth Medium with the provided supplements (Lonza). Conversely, CL1-0, CL1-5, H1975, and H3255 were maintained in RPMI 1640 with 10% FBS and 1% antibiotic-antimycotic. Colon normal FHC (ATCC CRL-1831) cells were sustained in DMEM/F12 medium (Gibco) supplemented with 10% FBS, 10 ng/mL cholera toxin, 0.005 mg/mL insulin, 0.005 mg/mL transferrin (Sigma), 100 ng/mL hydrocortisone, 20 ng/mL human recombinant EGF, and 1% antibiotic-antimycotic. In contrast, cancer epithelial cells SW620 (ATCC CCL-227) and SW1116 (ATCC CCL-233) were cultured in Leibovitz's L-15 medium with 10% FBS and 1% antibiotic-antimycotic. DLD-1 (ATCC CCL-221) and Colo205 (ATCC

CCL-222) were grown in ATCC-formulated RPMI 1640 (Gibco) with 10% FBS and 1% antibiotic-antimycotic. HT29 (ATCC HTB-38) cells were cultured in McCoy's 5a medium (Gibco) with 10% FBS and 1% antibiotic-antimycotic. All mammalian cell lines were cultured at 37°C with 5% CO2, with the exception of Expi293 cells cultured at 8% CO2 and colon cancers SW1116 and SW620 maintained in an air environment. Insect cells were cultured at 28°C without CO_2 incubation.

Plasmid Construction and Mutagenesis

The membrane-truncated human β3GalT5 was codon-optimized and decorated with a six-polyhistidine tag at the C-terminus for various expression hosts. This construct was synthesized by GenScript. All necessary restriction enzymes and the NEBuilder® HiFi DNA Assembly Cloning Kit for cloning were obtained from NEB, with the exception of the QIAquick™ gel extraction kit from Qiagen. For *E. coli* expression, the plasmid pET28a-TF-β3GalT5 was constructed by assembling BamHI-digested pET28a (Novagen), trigger factors, and β3GalT5. The creation of the bacmid pBAC-β3GalT5 for insect cell expression involved using EcoRI-digested pBAC-1 (Novagen) and PCR-amplified β3GalT5. To construct the plasmid for HEK293 cells, pHEK293 Ultra expression vector I was digested with XhoI and then ligated with β3GalT5. For generating mutations at positions S66 and Q69, as well as other catalytic residues, we followed a published method. [2] In summary, KOD Fx Neo (TOYOBO), mutated primers, and the wild type β3GalT5 sequence were added to a PCR reaction. The resulting PCR product was digested with DpnI for at least one hour and then transformed into DH5α competent cells. The mutated transformants were confirmed by sequencing. The S66A mutant primer sequences paired were forward: 5’-CTCGTGCTGCTGGTCACCTCCGCCCATAAGCAG-3’ reverse: 5’-GCTCGGCGAGCTGCTTATGGGCGGAGG TGAC-3’; Q69A mutant forward: 5’-CTGCTGGTCACCTCCTCCCATAAGGCCCTCGCCGAG-3’ reverse: 5’-CTAATGGCCATGCGCTCGGCGAGGGCCTTATGGGAG-3’; Q69M mutant forward: 5’- CTGCTGGTCACCTCCTCCCATAAGATGCTCGCCGAG-3’, reverse: 5’- CTAATGGCCATGCGCTCGGCGAGCATCTTATGGGAG-3’.

Protein Expression and Analysis

For bacterial expression of recombinant TF-β3GalT5 and other enzymes used in Globo-series glycans synthesis, an overnight culture of BL21(DE3), or enhanced protein folding strains like Origami B(DE3) (Novagen) and SHuffle T7 (NEB), was inoculated at a 1:50 (v/v) ratio into 1-liter Terrific Broth (Sigma) in a 2.5L flask (Thomson). The cells were induced with 0.4mM IPTG when the OD600 reached 0.8 to 1.0 and were incubated at 16 °C for 24 hours. After harvesting the cell pellet and lysing it with Bugbuster Protein Extraction reagent (Millipore), the supernatant containing the target proteins with a 6x-His tag was passed through an IMAC column (Cytiva). The proteins

were further washed and eluted with eight column volumes of 50mM MOPS pH 7.5, 300 mM NaCl, and either 20 mM or 200 mM imidazole. Each fraction was analyzed by SDS-PAGE with Coomassie Blue staining and confirmed by Western blot using rabbit anti-β3GalT5 polyclonal antibody (Abgent) or ABclonal mouse anti-His tag mAb (ABclonal Inc., USA). The enzymes were concentrated using an AmiconUltra with an appropriate molecular weight cutoff (Millipore) and their concentrations were quantified using the BCA Protein Assay Kit (Pierce). β3GalT5 was further purified using a size exclusion column (Superdex 200 Increase 10/300 GL, Cytiva) to obtain pure proteins. For the insect cell expression system, P1 baculovirus production followed the user guide for ExpiFectamine Sf Transfection Reagent (Gibco). The titer of baculovirus was determined using serial dilutions and infecting Sf-9 ET cells, measuring the GFP signal. During protein expression, 1.5 to 2.0 million per mL Sf-9 cells were infected with P2 virus at a 1:50 to 1:100 (v/v) ratio and harvested after 96 hours of infection. The purification procedure was the same as for *E. coli,* except the buffer system was changed to HEPES buffer, and an additional wash with two column volumes of 50 mM imidazole was performed before elution. To produce β3GalT5 in Expi293 mammalian cells, the ratio of ExpiFectamine 293 reagent (Gibco) to plasmid followed the manufacturer's instructions, with modification of harvest time (48 to 72 hours) and addition of HEPES (50 mM) 24h post-transfection without enhancers. The purification procedure for β3GalT5 in Expi293 cells was the same as that for insect cells mentioned above.

X-Ray Crystal Structure, Enzymatic Kinetics, Activity and protein thermostability Measurement.

The activity of β3GalT5 was assessed using the UDP-Glo assay (Promega), which quantifies the amount of UDP generated during the enzymatic reaction. Since this assay kit was designed to measure the hydrolysis of UDP-Gal with water as acceptor and did not contain any glycan as acceptor, we added a glycan acceptor to the assay mixture to measure the glycosyltransferase reaction to the glycan acceptor. Briefly, the enzymatic mixture containing 20 mM HEPES (pH 7.4), 0.1 mM $MnCl_2$, 0.5μM of recombinant β3GalT5, 5mM Gb4 glycan or other acceptors and 0.1 mM UDP-galactose (Promega) was incubated at 37°C for one hour with gentle shaking. After incubation, the reaction was quenched by same volume of UDP detecting reagent prepared from kit, and the luminescent signal was measured using a microplate reader CLARIOstar (BMG LABTECH). For substrate specificity, disaccharide acceptors, glycolipids and N glycans derivatives list on Table 1 were majorly purchased from BIOSYNTH™, except O glycans from Tokyo Chemical Industry Co., Ltd and agalacto-SGP from Fushimi Pharmaceutical Co., Ltd. To determine the kinetic parameters of β3GalT5 and its mutants, different concentrations of UDP-galactose or Gb4 glycan were diluted and

measured. The results were fitted using the Michaelis-Menten model, and the Km and kcat values were determined using GraphPad Prism 10. The detailed x-ray crystal structure and mechanistic study of β3GalT5 was submitted for publication and is under revision. To determine the melt temperature (Tm) of WT and S66A β3GalT5, 1 μg of each protein was mixed with Protein Thermal Shift™ dye (Applied Biosystems) in six replicates, following the manufacturer's instructions. The reactions were then heated and monitored using the QuantStudio™ 5 Real-Time PCR System. Data analysis was performed with Protein Thermal Shift™ Software v1.4 (Applied Biosystems).

Synthesis of UDP-galactose analogs, UDP-Gal-6-aldehyde and UDP-Gal-6-azide.

To synthesize UDP-galactose-6-aldehyde, a 4 mL solution containing 22 mg UDP-galactose was oxidized directly using 500 μg/mL galactose oxidase (Sigma), 50 μg/mL horseradish peroxidase (Sigma), and 75 μg/mL catalase in a 100 mM sodium phosphate buffer (pH 7.4). The oxidation process occurred at room temperature for three hours with gentle shaking. The reaction was facilitated by the addition of a trace amount of hydrogen peroxide (Sigma), and the resulting product was first passed through 10K AmiconUltra to remove proteins, then subsequently purified using Bio-Gel P-2 Gel (Bio-rad). For the synthesis of UDP-galactose-6-azide, a 4.8 mL mixture containing 5 mg 6-azido-galactose (BIOSYNTH™) was combined with 5 mM UTP, 6.25 mM ATP and 10 mM $MgCl_2$ in a 50 mM Tris-HCl buffer (pH 7.2). Then, 0.1 mM *Pisum sativum* UDP-sugar pyrophosphorylase (PsUSP), 0.5mM PPA, and 0.5mM *Bifidobacterium infantis* galactokinase (BiGalK L401H) were added to the mixture and the reaction was continued at 42 °C for five hours with gentle shaking. The product was subsequently purified using Bio-Gel P-2 Gel (Bio-rad), and the structure of the product was confirmed with mass spectrometry and NMR analysis.

Quantitative PCR and droplet-digital PCR.

RNA samples were extracted from both normal and cancer cells using RNeasy Kits (Qiagen) and subsequently converted into cDNA using SuperScript™ III Reverse Transcriptase (Invitrogen). To detect the isoforms β3GalT5-1 and β3GalT5-2, we designed specific probes and primer sets tailored to each variant. For isozyme 1, the forward primer sequence is 5'-CCAAGCCCAGAACCTGATAAT-3', the reverse primer is 5'-AGAGGAACCTCTGGTTTGAAAG-3', and the probe sequence is 5'-/56-FAM/TCCTCTTGGCATTTACACTGTGGCT/3IABkFQ/. For isozyme 2, the forward primer sequence is 5'-GAGAAACTGCCATTCCGTTATTG-3', the reverse primer is 5'-CCCAGAACCAGAAGGCAAATA-3', and the probe sequence is 5'-/56-FAM/TGTGTTTGGAGATGGCTTTCCCGA/3IABkFQ/ (Integrated DNA Technologies). To assess the efficiency of knockdown through real-time PCR, we combined cDNA, targeting probes, and KAPA PROBE FAST Master Mix (Roche), and then conducted the detection using QuantStudio 5. The amplification results were

further processed to calculate relative expression using the delta-delta Ct method. For absolute quantification of β3GalT5 isozymes in both normal and cancer cells via droplet-digital PCR (ddPCR), we followed the sample preparation steps, oil droplet formation, and target gene amplification protocols as per the instructions provided with the QX200 Droplet Digital PCR System. Subsequently, the droplets containing positive and negative signals were analyzed using the QX200 Droplet Reader and the QuantaSoft Pro software.

Preparation of glycosphingolipids (GSLs) and release of glycan from GSLs for labeling with NAIM Tag

As described previously[3], cells were harvested, washed with PBS, and homogenized in water. The homogenate was then mixed with methanol and chloroform to the ratio of water:methanol: chloroform=3:8:4 (v/v/v) and incubated in a bath sonicator for 30 minutes. After centrifugation at 12,000 x g for 1 minute, the pellet underwent repeated extraction with a water/methanol/chloroform mixture at a ratio of 3:8:4 (v/v/v). The combined supernatant was dried using a SpeedVac. Folch Partition was performed by dissolving the dried sample in chloroform and methanol at a ratio of 2:1 (v/v). Next, 0.1 M NaCl was added to the sample, which was then centrifuged at 12,000 x g for 1 minute. The upper layer was collected, and the same volume of methanol and 0.1 M NaCl was added to the sample before repeating the centrifugation process twice. Finally, all collections were combined in a glass bottle. The GSLs were treated with ozone in methanol in a glass bottle until a blue color appeared. Two drops of methyl sulfide (Me_2S) were added to the resulting solution and stirred at room temperature for 2 hours. Then, 0.5 M NaOH (final concentration 50 mM) was added to the sample and stirred overnight. After the reaction, the sample was dried using a rotary evaporator and cleaned up using C18 and PGC spin columns. After being released from GSLs, the glycan mixture was lyophilized and labeled using established literature procedures. In brief, the glycan was mixed with 2,3-naphthalenediamine (NAIM, 1.0 mg) and iodine (1.0 mg) in AcOH (1.0 mL) at room temperature and stirred overnight. The resulting reaction mixture was then triturated with EtOAc (10.0 mL × 2) to obtain precipitates for MS analysis.

LC-MS/MS analysis

Samples were detected by LC-ESI-MS on an Orbitrap Fusion mass spectrometer (Thermo Fisher Scientific, San Jose, CA) equipped with Waters ACQUITY UPLC M-Class (Waters, Milford, MA) and EASY-spray source (Thermo, San Jose, CA, US). The sample solution was injected (5 µL) at a flow rate of 7 µL/min onto a precolumn (C18, 0.180 mm x 20 mm, ID 5 µm; Waters). Chromatographic separation was performed on an easy column (C18, 0.075 mm x 150 mm, ID 3 µm; Thermo Scientific) using 0.1% formic acid in water as mobile phase A and 0.1% formic acid in 100%

acetonitrile as mobile phase B, operated at a flow rate of 300 nL/min. Briefly, the gradient employed was from 1% buffer B to 40% buffer B at 44 min. The full-scan MS condition was a mass range of m/z 200-1800 (AGC target 5E5) with lock mass, resolution of 120,000 at m/z 200, and maximum injection time of 50 ms. The MSMS was run in top speed mode with 1s cycles with HCD, while the dynamic exclusion duration was set to 60 s with a 10-ppm tolerance around the selected precursor and its isotopes. Electrospray voltage was maintained at 1.8 kV, and the capillary temperature was set at 275 °C.

For the analysis of galactosylation products, substrates and UDP-sugars, samples were analyzed by LC-ESI-MS/MS using a Velos Pro mass spectrometer (Thermo Fisher Scientific, San Jose, CA) coupled with an Ultimate 3000 RSLC system (Dionex Corporation, Sunnyvale, CA) and a standard ESI ion source. A porous graphitic carbon (PGC) column (Hypercarb, 1.0 mm × 150 mm, 3 µm particle size; Thermo Fisher Scientific) was employed for separation. The gradient program involved an increase from 5% buffer B at 5 minutes to 40% buffer B at 39 minutes, with a flow rate of 50 µL/min. Buffer A consisted of 0.1% formic acid in water, and buffer B was 0.1% formic acid in 80% acetonitrile. Full-scan MS conditions included a mass range of m/z 200–2000 with enhanced resolution. Targeted m/z ions were sequentially isolated and fragmented using CID with rapid scan mode. The electrospray voltage was set to 4.0 kV, and the capillary temperature was maintained at 275°C.

Supplementary Figures and Tables

A.

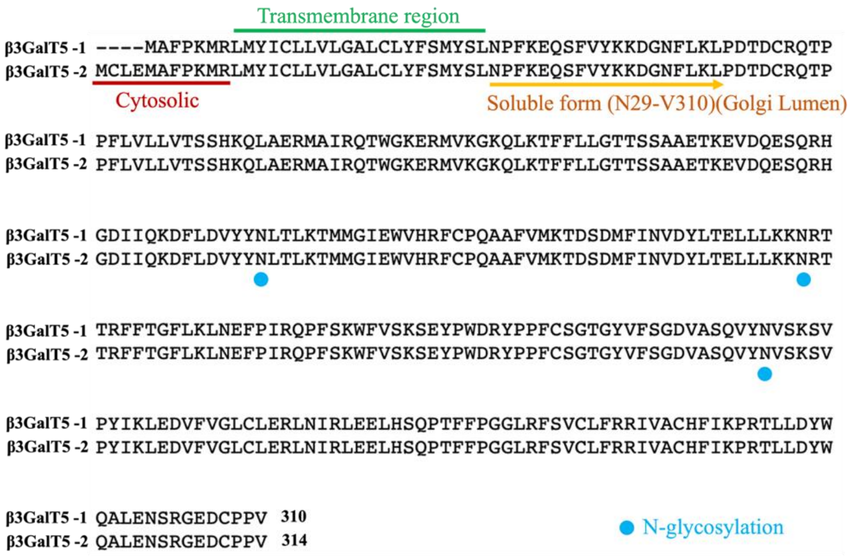

B.

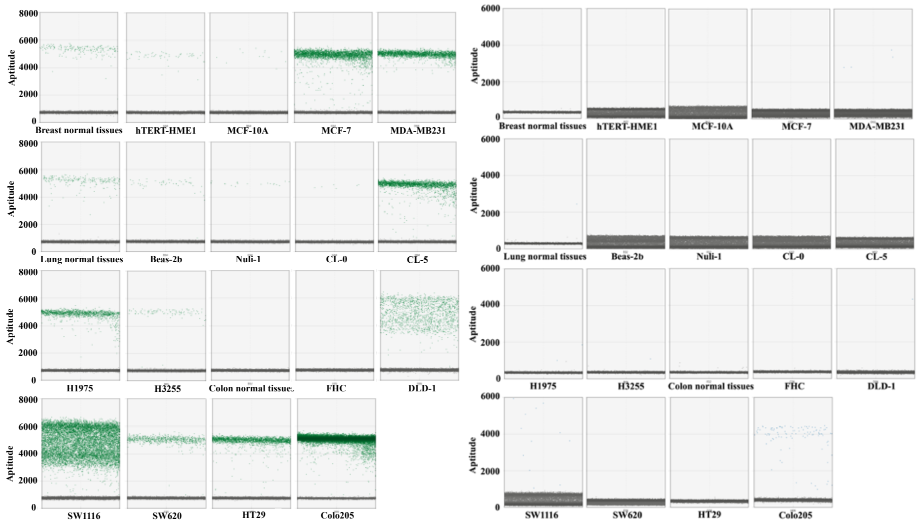

C.

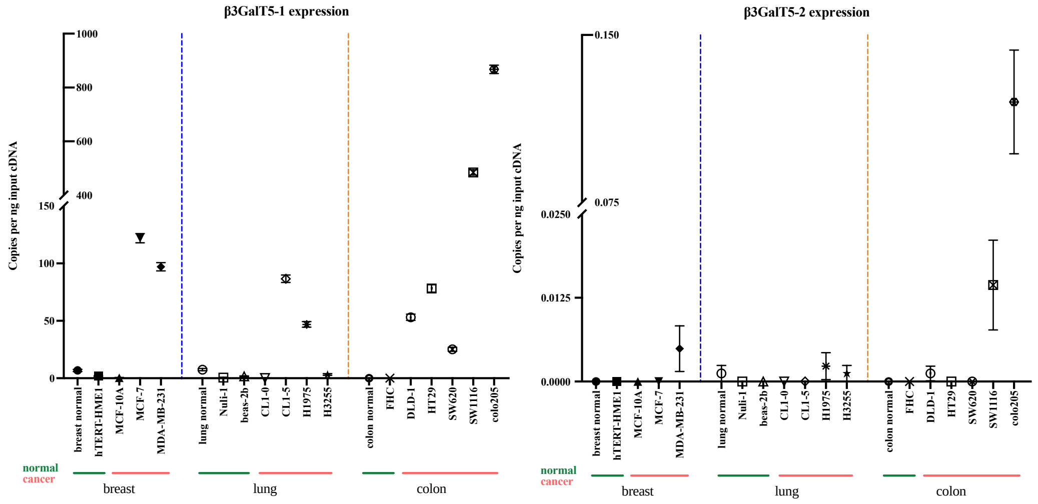

Figure S1: Protein and RNA sequences of isozymes β3GalT5-1 and β3GalT5-2.
(A) Alignment of β3GalT5-1 and β3GalT5-2 protein sequences. β3GalT5-2 contains additional four amino acids in the N-terminal cytosol region. The soluble form of β3GalT5 (N29-V310) including the catalytic domain is located at the Golgi lumen. The blue dots indicated the N-glycosylation sites. (B) Distribution of β3GalT5-1 (green dots as positive droplets) and β3GalT5-2 (gray dots as negative droplets) in normal and cancer cells. (C) Expression profile of two human β3GalT5 isozymes in normal and cancer cells. The left panel illustrates the RNA expression of β3GalT5-1, while the right panel illustrates the RNA expression of β3GalT5-2.

Table S1. dsiRNA sequences designed to target β3GalT5-1.

Name	dsiRNA Duplex Sequences (β3GalT5-1)
dsiRNA #1	5'-rArArCrCrUrGrArUrArArUrUrArUrGrGrArGrCrArUrUrCTA-3'<br>5'-rUrArGrArArUrGrCrUrCrCrArUrArArUrUrArUrCrArGrGrUrUrCrU-3'
dsiRNA #2	5'-rArArCrCrArArGrCrCrCrArGrArArCrCrUrGrArUrArArUTA-3'<br>5'-rUrArArUrUrArUrCrArGrGrUrUrCrUrGrGrGrCrUrUrGrGrUrUrUrG-3'
dsiRNA #3	5'-CrCrUrGrArUrArArUrUrArUrGrGrArGrCrArUrUrCrUrACA-3'<br>5'-rUrGrUrArGrArArUrGrCrUrCrCrArUrArArUrUrArUrCrArGrGrUrU-3'
dsiRNA #4	5'-rArArArCrCrArArGrCrCrCrArGrArArCrCrUrGrArUrArATT-3'<br>5'-rArArUrUrArUrCrArGrGrUrUrCrUrGrGrGrCrUrUrGrGrUrUrUrGrU-3'
dsiRNA #5	5'-rCrUrGrArUrArArUrUrArUrGrGrArGrCrArUrUrCrUrArCAC-3'<br>5'-rGrUrGrUrArGrArArUrGrCrUrCrCrArUrArArUrUrArUrCrArGrGrU-3'
dsiRNA #6	5'-rArCrCrUrGrArUrArArUrUrArUrGrGrArGrCrArUrUrCrUAC-3'<br>5'-rGrUrArGrArArUrGrCrUrCrCrArUrArArUrUrArUrCrArGrGrUrUrC-3'
dsiRNA #7	5'-GrArArCrCrUrGrArUrArArUrUrArUrGrGrArGrCrArUrUCT-3'<br>5'-rArGrArArUrGrCrUrCrCrArUrArArUrUrArUrCrArGrGrUrUrCrUrG-3'
dsiRNA #8	5'-rArUrArArUrUrArUrGrGrArGrCrArUrUrCrUrArCrArCrUGA-3'<br>5'-rUrCrArGrUrGrUrArGrArArUrGrCrUrCrCrArUrArArUrUrArUrCrA-3'
dsiRNA #9	5'-GrArUrArArUrUrArUrGrGrArGrCrArUrUrCrUrArCrArCTG-3'<br>5'-rCrArGrUrGrUrArGrArArUrGrCrUrCrCrArUrArArUrUrArUrCrArG-3'
dsiRNA#10	5'-rArCrCrArArGrCrCrCrArGrArArCrCrUrGrArUrArArUrUAT-3'<br>5'-rArUrArArUrUrArUrCrArGrGrUrUrCrUrGrGrGrCrUrUrGrGrUrUrU-3'
dsiRNA #11	5'-rGrArUrArGrUrUrArCrArCrCrArUrUrGrGrCrArUrArUrCTG-3'<br>5'-rCrArGrArUrArUrGrCrCrArArUrGrGrUrGrUrArArCrUrArUrCrArG-3'

A.

B.

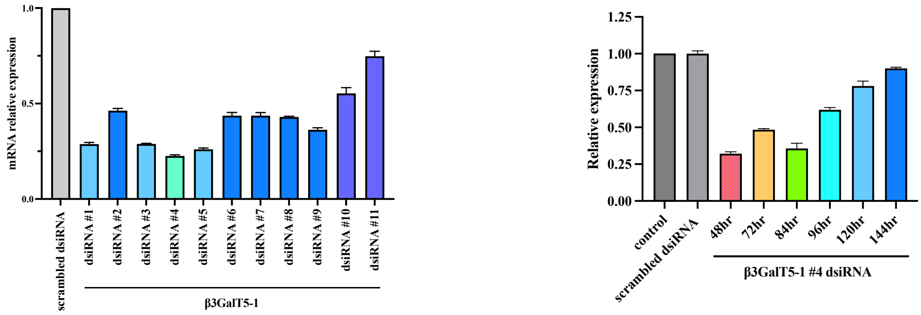
mRNA relative expression
scrambled dsiRNA
dsiRNA #1
dsiRNA #2
dsiRNA #3
dsiRNA #4
dsiRNA #5
dsiRNA #6
dsiRNA #7
dsiRNA #8
dsiRNA #9
dsiRNA #10
dsiRNA #11
β3GalT5-1
Relative expression
control
scrambled dsiRNA
48hr
72hr
84hr
96hr
120hr
144hr
β3GalT5-1 #4 dsiRNA

C.

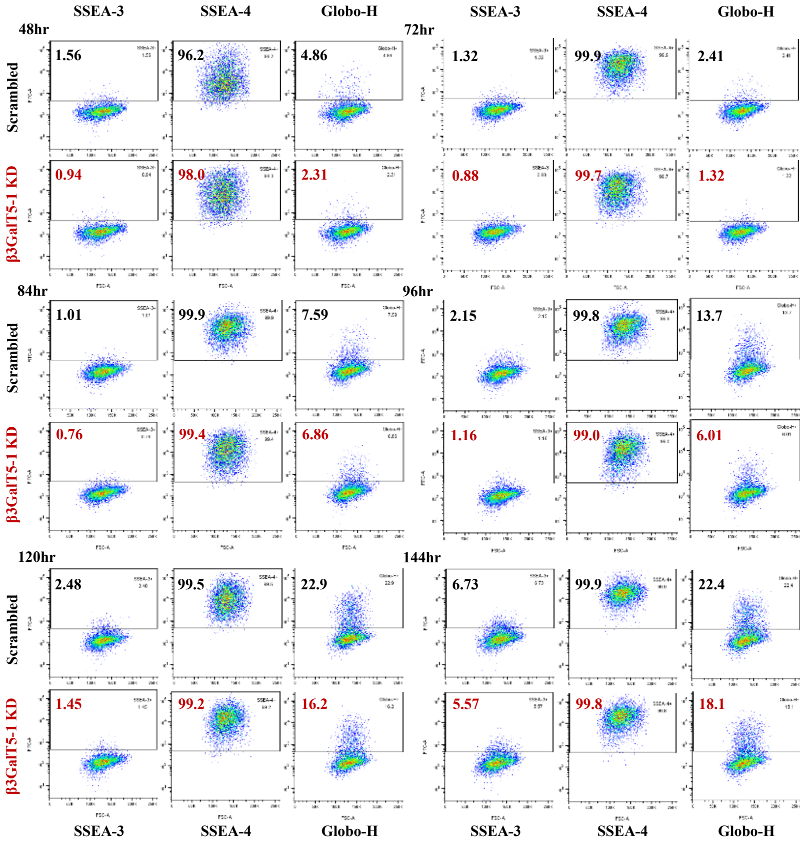
SSEA-3
SSEA-4
Globo-H
48hr
72hr
84hr
96hr
120hr
144hr
Scrambled
β3GalT5-1 KD

D.

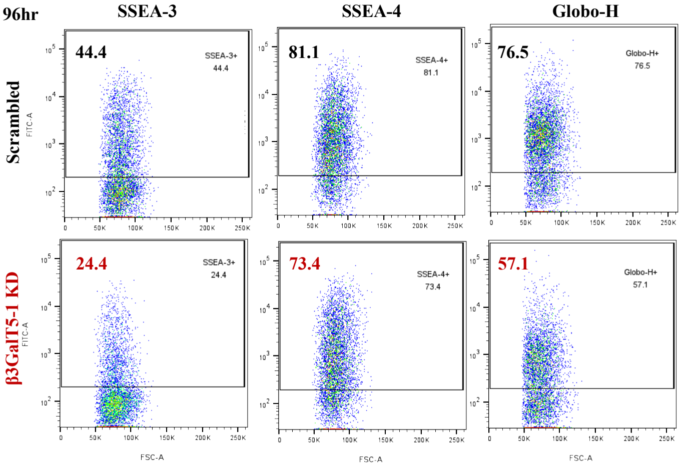
96hr
SSEA-3
SSEA-4
Globo-H
Scrambled
β3GalT5-1 KD
44.4
81.1
76.5
24.4
73.4
57.1
FSC-A

E.

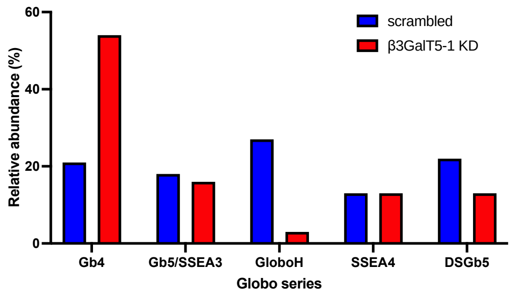

scrambled
β3GalT5-1 KD
Relative abundance (%)
60
40
20
0
Gb4
Gb5/SSEA3
GloboH
SSEA4
DSGb5
Globo series

F.

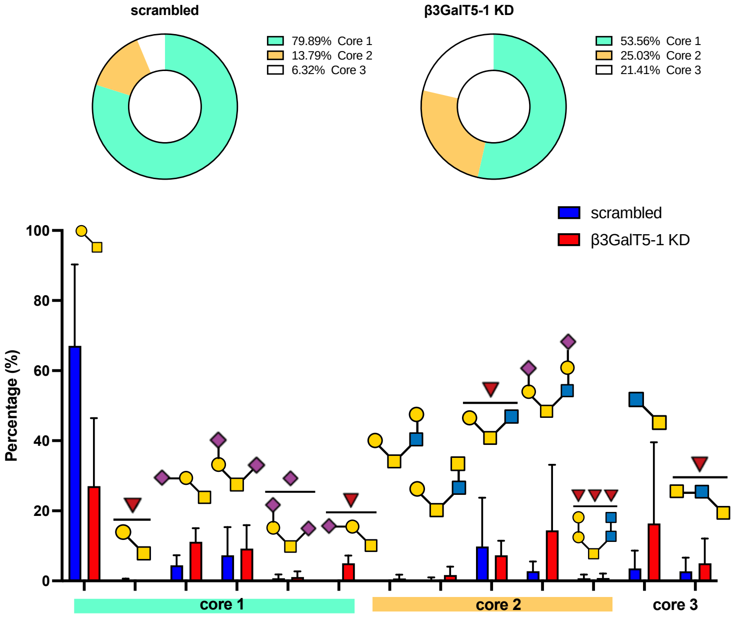

scrambled
79.89% Core 1
13.79% Core 2
6.32% Core 3
β3GalT5-1 KD
53.56% Core 1
25.03% Core 2
21.41% Core 3
scrambled
β3GalT5-1 KD
Percentage (%)
100
80
60
40
20
0
core 1
core 2
core 3

G.

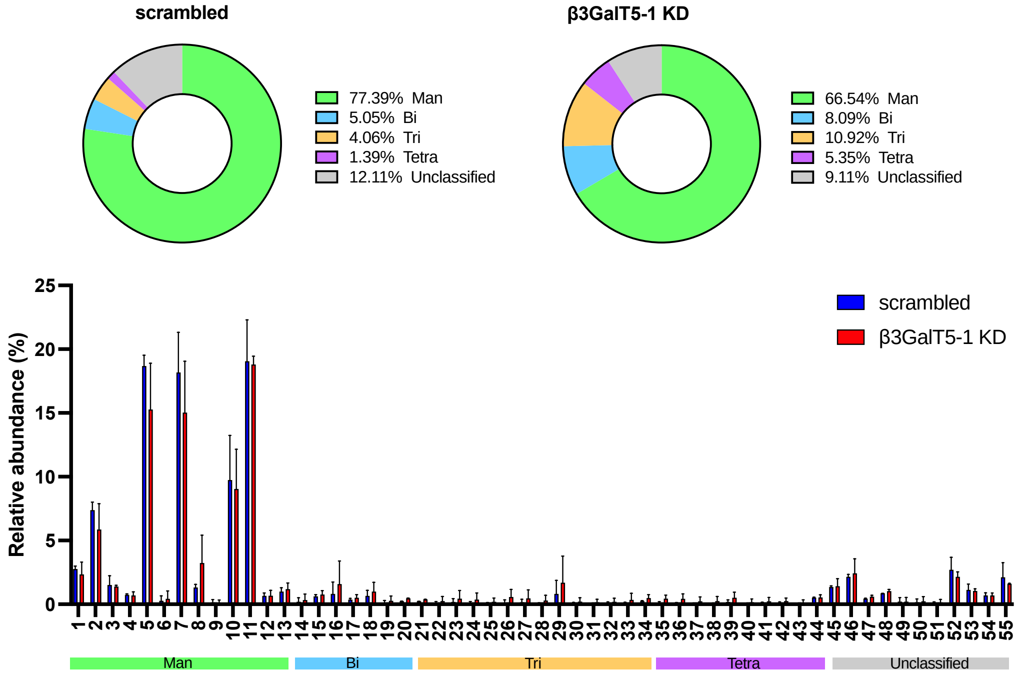

scrambled
77.39% Man
5.05% Bi
4.06% Tri
1.39% Tetra
12.11% Unclassified
β3GalT5-1 KD
66.54% Man
8.09% Bi
10.92% Tri
5.35% Tetra
9.11% Unclassified
scrambled
β3GalT5-1 KD
Relative abundance (%)
25
20
15
10
5
0
Man
Bi
Tri
Tetra
Unclassified

Figure S2. Glycosylation changed after knocking down of β3GalT5-1 in breast cancer cells. (A) Eleven dicer-substrate siRNAs (dsiRNAs) designed to target β3GalT5-1 isozyme were evaluated and analyzed by qPCR. (B) Relative expression levels of β3GalT5-1 after dsiRNA-mediated knockdown with #4 dsiRNA. (C) The cell surface expression levels of SSEA-3, SSEA-4, and Globo-H in MDA-MB231 were analyzed by flow cytometry at various time points after knockdown of β3GalT5-1 with #4 dsiRNA. Scrambled indicated non-silencing and negative control dsiRNA that do not recognize any sequences in human transcriptomes. (D) The cell surface SSEA-3, SSEA-4, and Globo-H expression on MCF-7 cells after knocking down β3GalT5-1 by #4 dsiRNA at 96 hrs. (E) The Globo-series glycosphingolipids distribution between scrambled (blue bars) and β3GalT5-1 knockdown cells (red bars) after knocking down β3GalT5-1 with #4 dsiRNA in MCF-7 cells. (F) Profiling of O-glycans after knocking down β3GalT5-1 with #4 dsiRNA in MCF-7 cells after 96 hours. The O-glycans were released from O-glycopeptide through β-elimination by sodium bromide and further purified by C-18 column and PGC column before injecting to mass spectrometry for analysis. The yellow and blue circles represent galactose and glucose, respectively. The yellow and blue square represent N-acetylgalactosamine and N-acetylglucosamine, respectively. Red triangle stands for fucose, and purple diamond stands for sialic acid. (G) N-glycans from MCF-7 cells after knockdown of β3GalT5-1 with #4 dsiRNA for 96 hours were released by PNGaseF and analyzed by mass spectrometry. The symbol F stands for fucose, S for sialic acid and the number after F/S stands for the number of fucose or sialic acid on glycans. For mannose type glycans, 1: Man3, 2: Man3F, 3: Man4, 4: Man4F1, 5: Man5, 6: Man5F1, 7: Man5S1, 8: Man6, 9: Man6P1, 10: Man6F1, 11: Man7, 12: Man8, 13: Man9. Biantennary glycans, 14: BiF1, 15: BiS1, 16: BiS1F1, 17: BiS2, 18: BiS2F1, 19: BiS1F3. Tri-antennary glycans, 20: TriF1, 21: TriF2, 22: TriS1F1, 23: TriS3, 24: TriS2, 25: TriS2F1, 26: TriF4, 27: TriS2F1, 28: TriS3F1, 29: TriS3, 310: TriS2F2, 31: TriS1F4, 32: TriS3F1, 33: TriS4. Tetra-antennary glycans, 34: TrtraF1, 35: TetraF2, 36: TetraS1F1, 37: TetraF3, 38: TetraS2F1, 39: TetraS2F1, 40: TetraS3F1, 41: TetraS2F2, 42: TrtraS3F1, 43: TrtraS2F3. Others glycans 44 to 55 not group above belong to hybrid type or unclassified glycans.

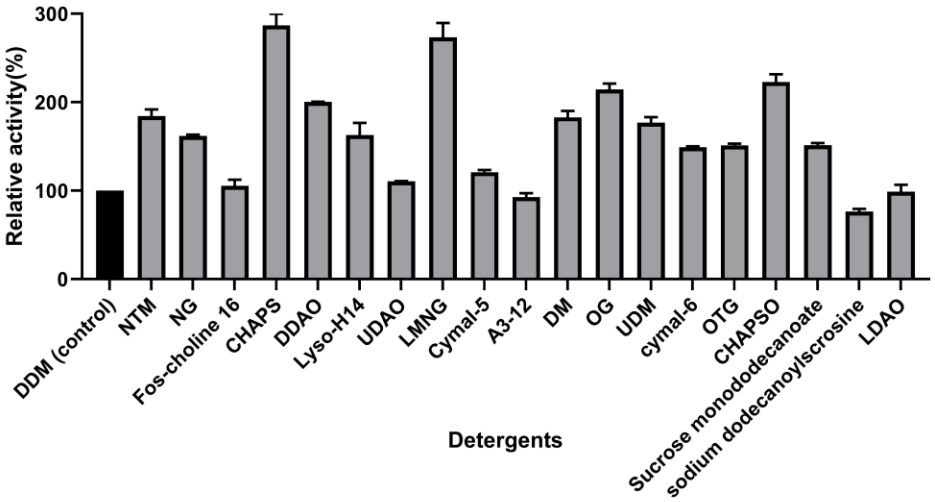

Figure S3. Detergent screening of β3GalT5-1 or β3GalT5-2 expressed from HEK293 cells. Membrane-bound β3GalT5-1 expressed from HEK293 was extracted and the activity with different detergents was assessed with UDP-glo assay. DDM: n-dodecyl β-D-maltoside, NTM: n-nonyl-β-D-thiomaltoside, NG: n-nonyl-beta-D-glucopyranoside, CHAPS: 3-((3-cholamidopropyl) dimethylammonio)-1-propanesulfonate, DDAO: n-decyl-N,N-dimethylamine-N-Oxide, Lyso-H14: 1-myristoyl-2-hydroxy-sn-glycero-3-phospho-(1'-rac-glycerol), UDAO: n-undecyl-N,N-dimethylamine-oxide, LMNG: lauryl maltose neopentyl glycol, Cymal-5: 5-cyclohexyl-1-pentyl-β-D-maltoside, DM: n-decyl-beta-maltoside, OG: octylglucopyranoside, UDM: n-undecyl-beta-maltoside, Cymal-6: 6-cyclohexyl-1-hexyl-β-D-maltoside, OTG: octylthioglucoside, CHAPSO: 3-[(3-cholamidopropyl)dimethylammonio]-2-hydroxy-1-propanesulfonate, LDAO: lauryldimethylamine-N-oxide.

A. Substrate 1-2

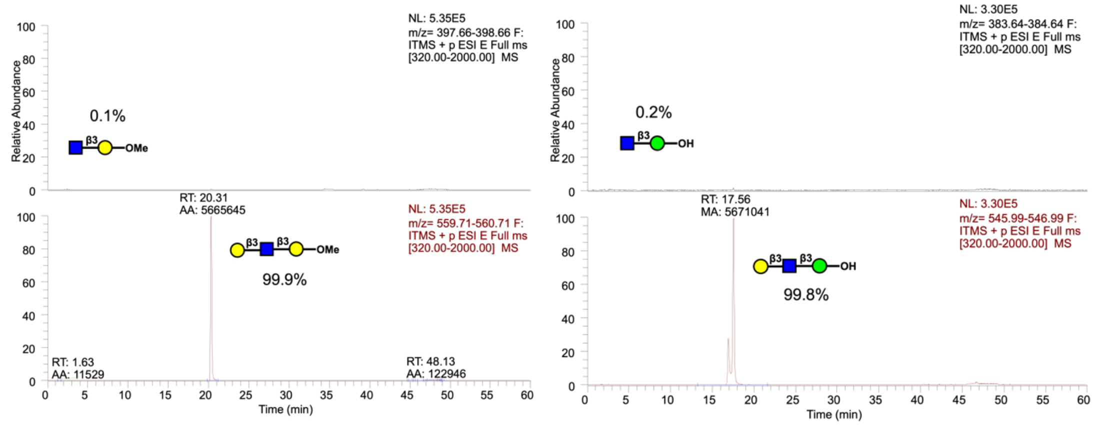

Substrate 3-4

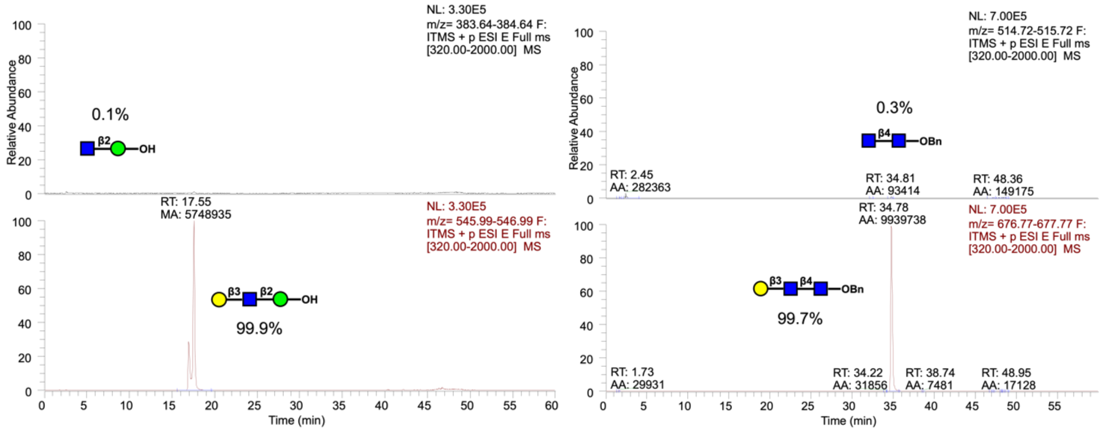

Substrate 5-6

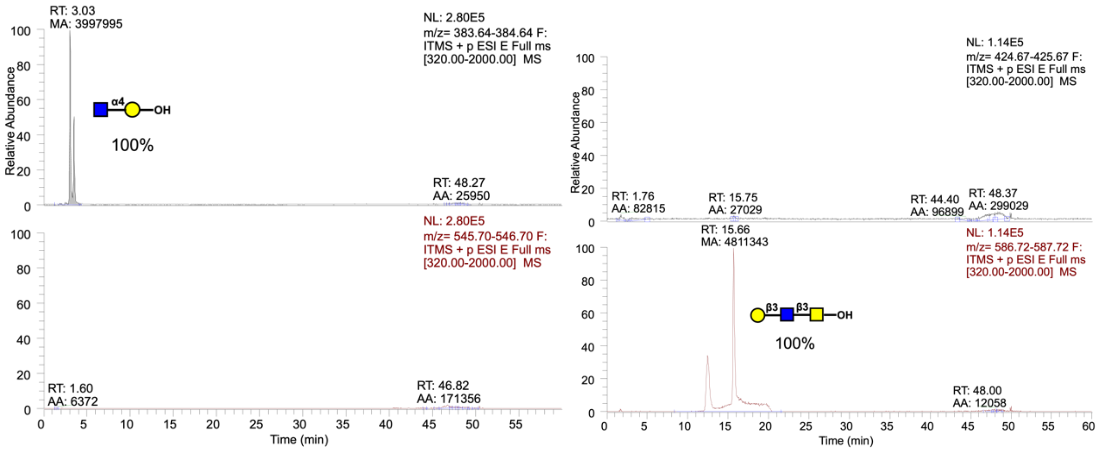

Substrate 7-8

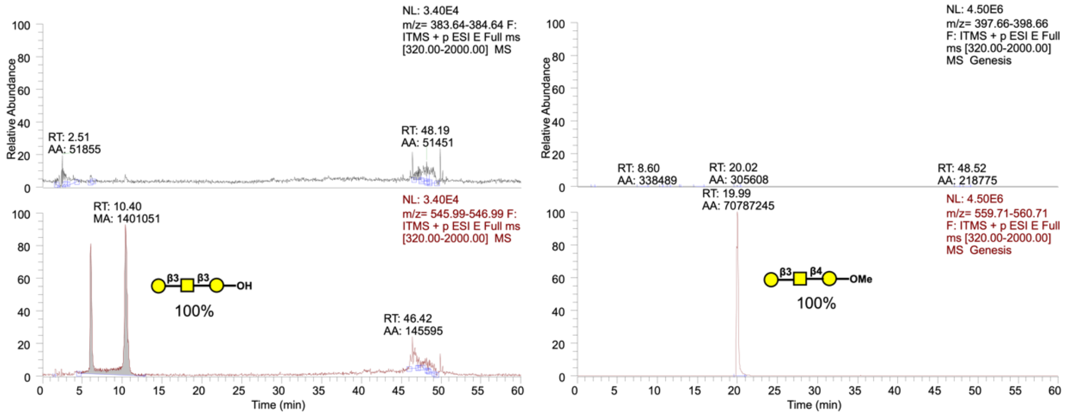

Substrate 9-10

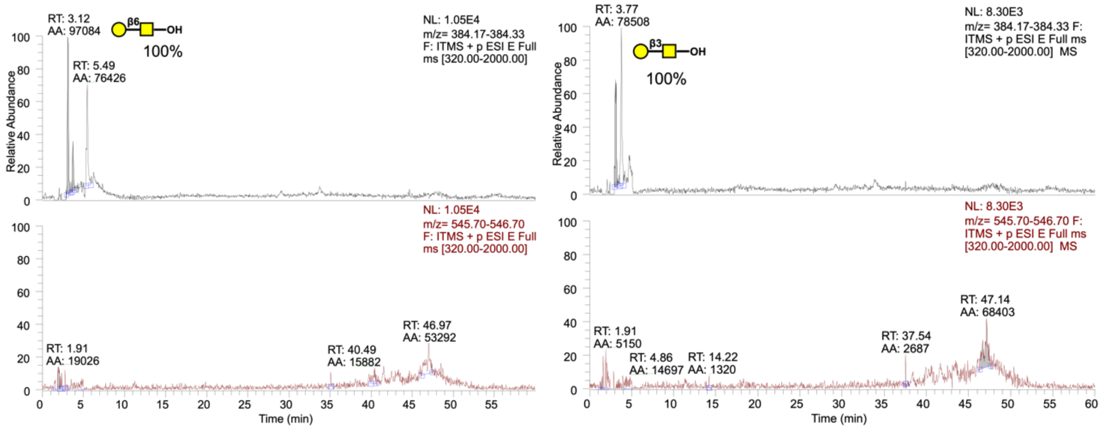

Substrate 11-12

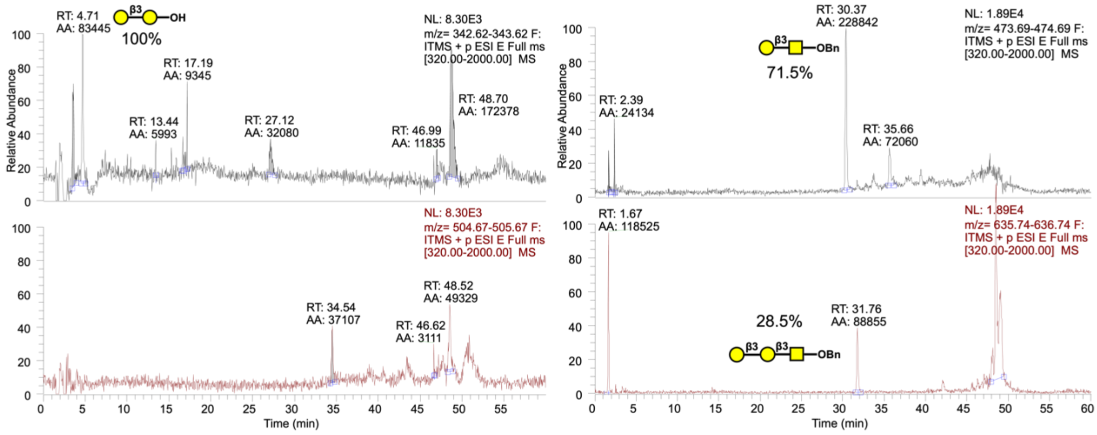

Substrate 13-14

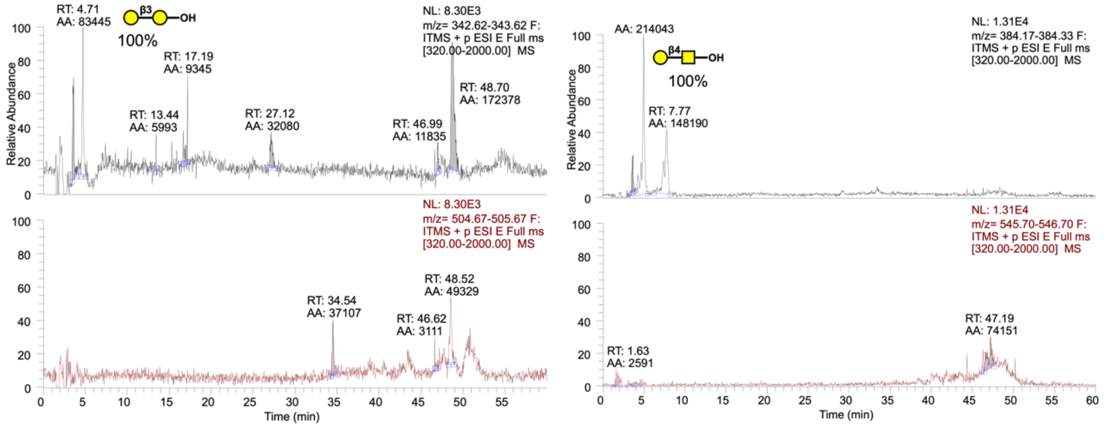

Substrate 15-16

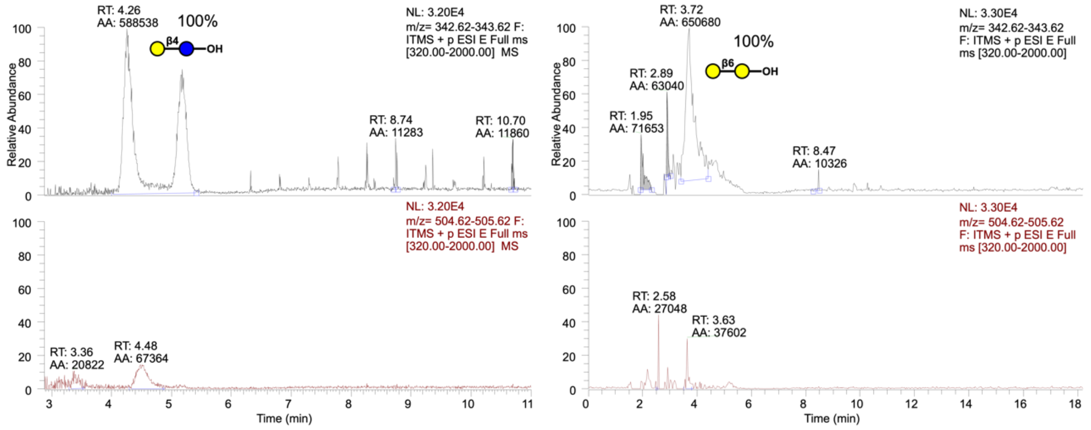

Substrate 17-18

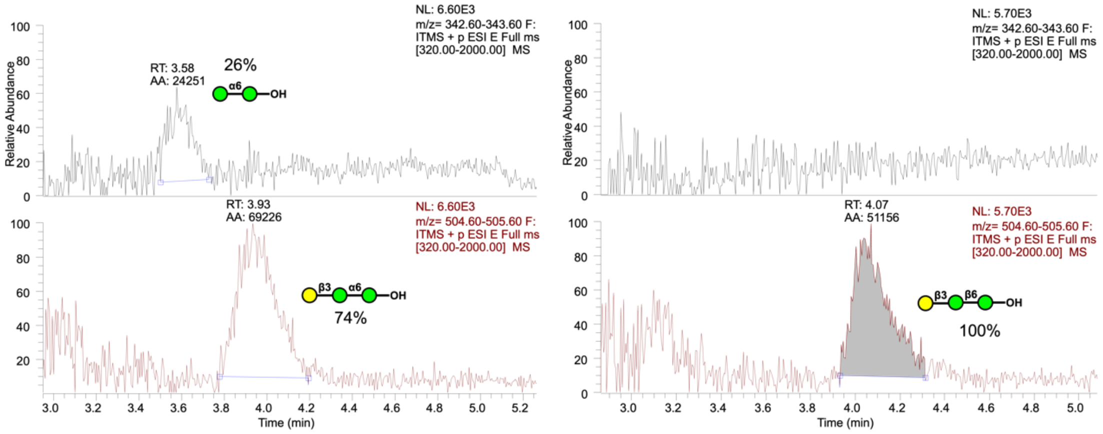

Substrate 19-20

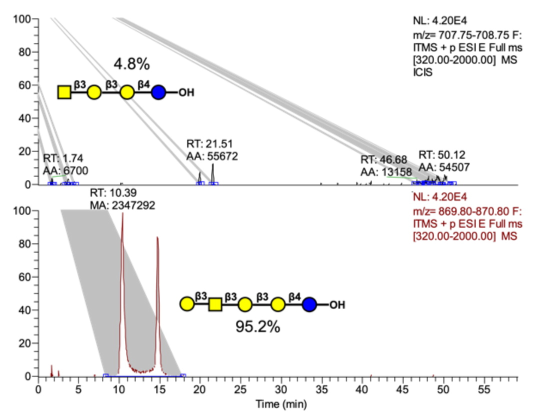

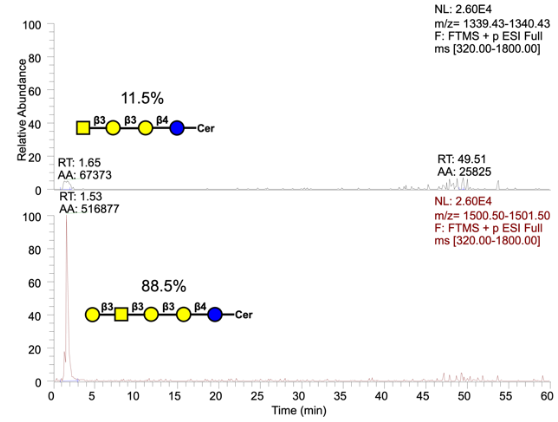

Substrate 21-22

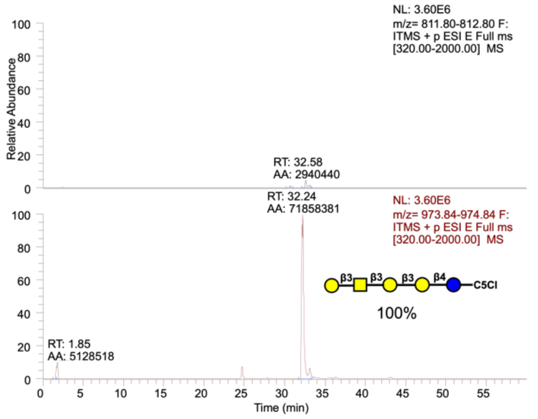

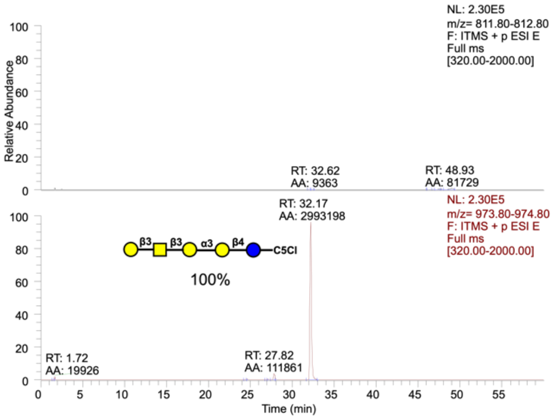

Substrate 23-24

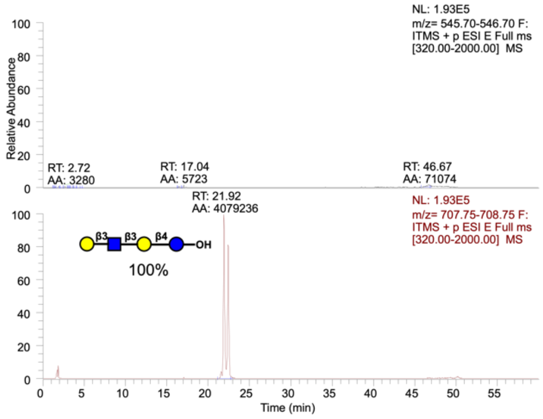

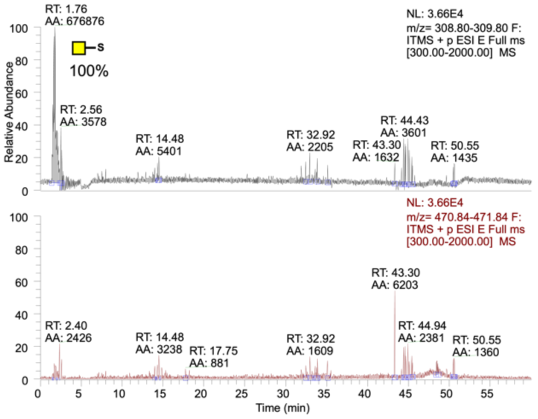

Substrate 25-26

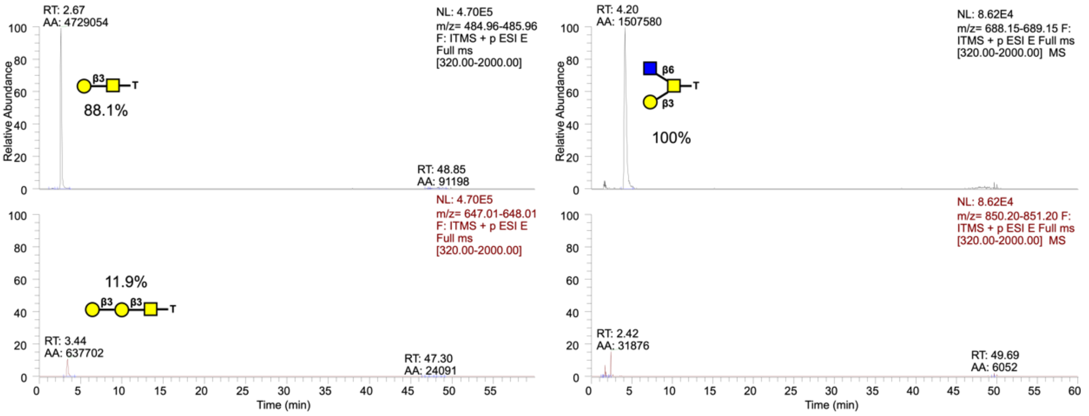

Substrate 27-28

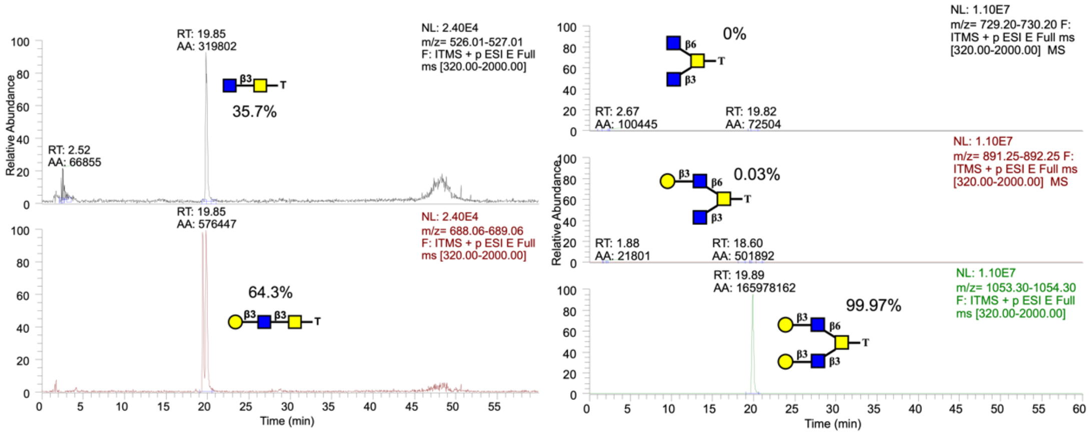

Substrate 29

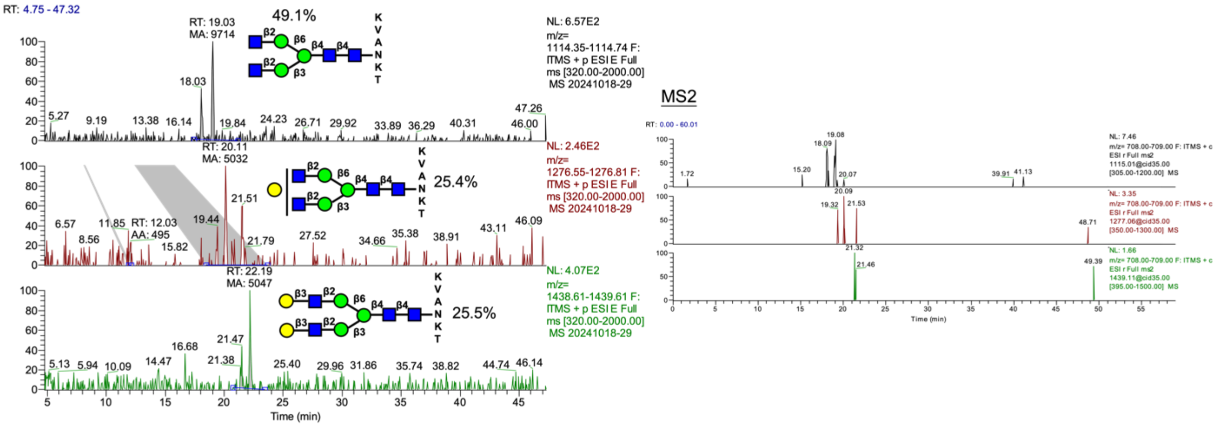

Substrate 30-31

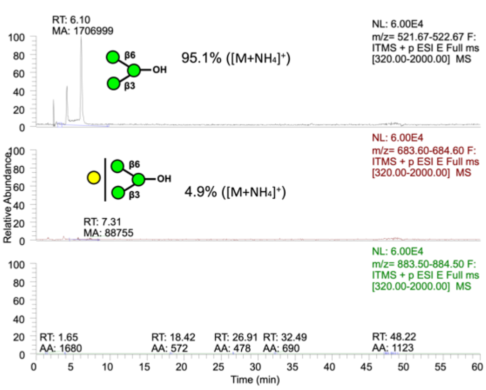

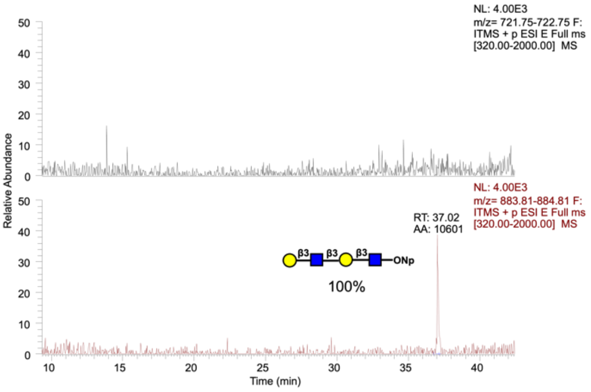

UDP-sugar analogs 1-2

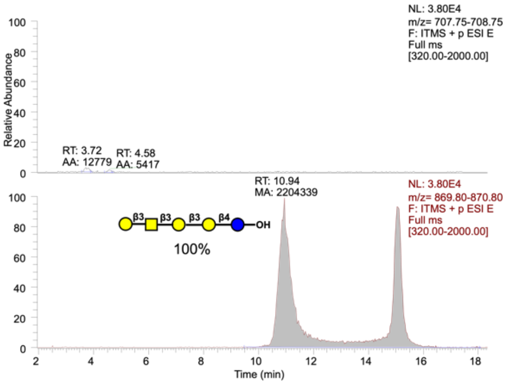

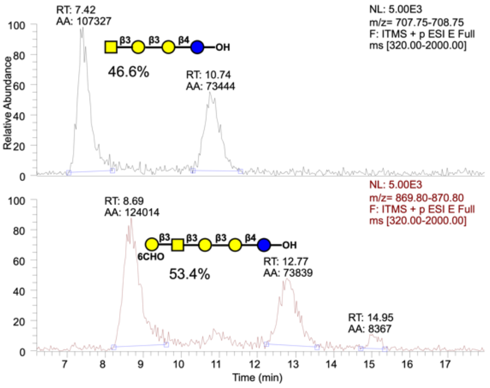

UDP-sugar analogs 3-4

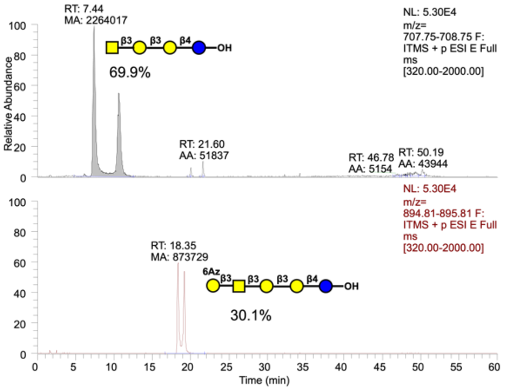

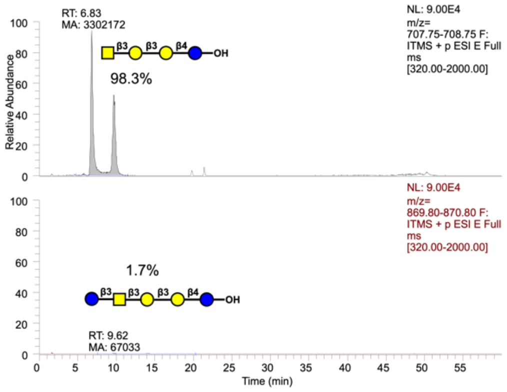

UDP-sugar analogs 5-6

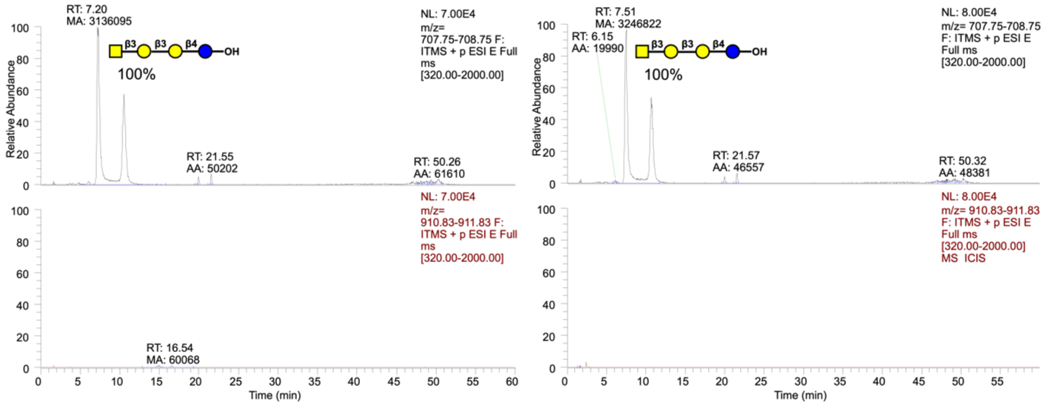

UDP-sugar analogs 7-8

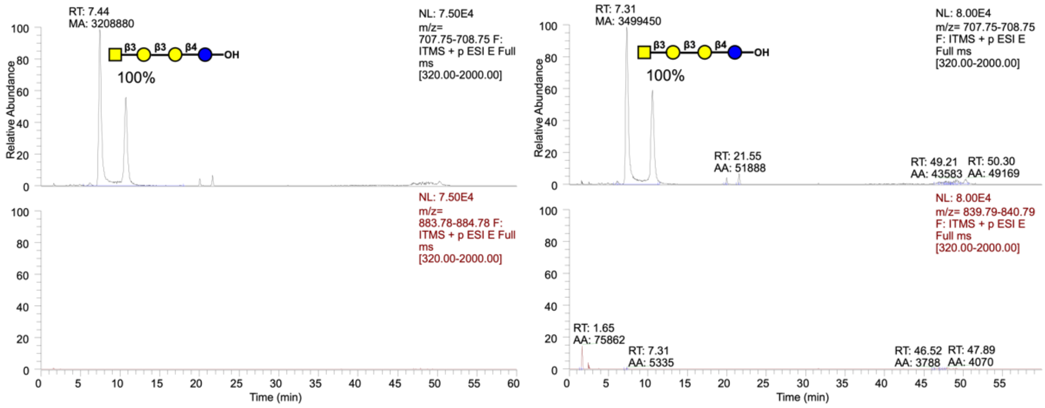

B.

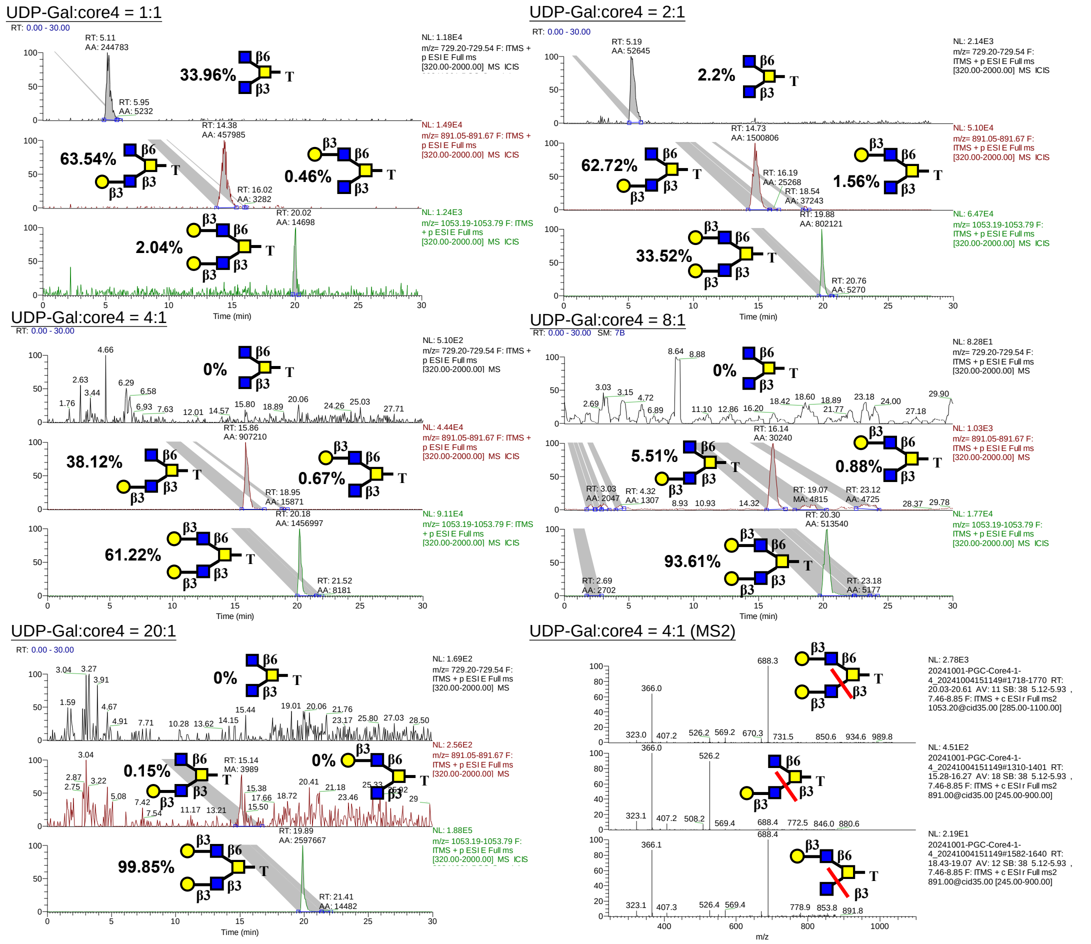

Figure S4. Products confirmation by LC-MS. (A) soluble β3GalT5-1 catalyzed galactosylation of glycan substrates with UDP-sugar donors. (B) O-linked core 4 structure was galactosylated by soluble β3GalT5-1 and analyzed by LC-MS.

Table S2. Galactosylation of O-linked core 4 structure through soluble β3GalT5.

UDP-Gal: core 4 molar ratio*	Core 4	Gal on β1,3 arm	Gal on β1,6 arm	Gal on both β1,3 and β1,6 arms
1:1	33.96%	65.4%	0.46%	2.04%
2:1	2.2%	62.72%	1.56%	33.52%
4:1	0%	38.12%	0.67%	61.22%
8:1	0%	5.51%	0.88%	93.61%
20:1	0%	0.15%	0%	99.85%

*Different ratios of galactose in UDP-Gal to O-glycan core 4 structure were incubated with recombinant soluble β3GalT5-1 from insect cells and the reaction mixture was analyzed by LC-MS after 24 hours at 37°C.

Figure S5. NMR spectrum of Allyl-Gb5, Gb5-C_5Cl, Gal-β1, 3-GlcNAc-β1, 3-Gal-OMe and Gal-β1, 3-Man-β1, 6-Man.

Allyl-Gb5

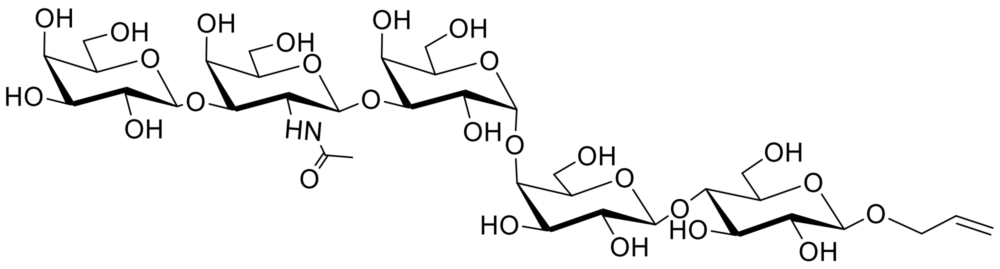

N-((2S,3R,4R,5R,6R)-2-(((2R,3R,4S,5S,6R)-2-(((2R,3R,4R,5R,6S)-6-(((2R,3S,4R,5R,6R)-6-(allyloxy)-4,5-dihydroxy-2-(hydroxymethyl)tetrahydro-2H-pyran-3-yl)oxy)-4,5-dihydroxy-2-(hydroxymethyl)tetrahydro-2H-pyran-3-yl)oxy)-3,5-dihydroxy-6-(hydroxymethyl)tetrahydro-2H-pyran-4-yl)oxy)-5-hydroxy-6-(hydroxymethyl)-4-(((2R,3R,4S,5R,6R)-3,4,5-trihydroxy-6-(hydroxymethyl)tetrahydro-2H-pyran-2-yl)oxy)tetrahydro-2H-pyran-3-yl)acetamide

1H NMR (600 MHz, D_2O) δ6.02-5.95 (m, 1H), 5.41-5.38 (dd, *J* = 17.2, 1.3 Hz, 1H), 5.30 (d, *J* = 10.5 Hz, 1H), 4.92 (d, *J* = 3.9 Hz, 1H), 4.70 (d, *J* = 8.5 Hz, 1H), 4.54 (d, *J* = 8.0 Hz 1H), 4.52 (d, *J* = 7.7 Hz, 1H), 4.46 (d, *J* = 7.7 Hz, 1H), 4.42-4.39 (m, 2H), 4.26-4.22 (m, 2H), 4.49 (d, *J* = 3.1 Hz, 1H), 4.09-3.90 (m, 8H), 3.86-3.58 (m, 17H), 3.55-3.52 (m, 1H), 3.35-3.33 (t, *J* = 8.6 Hz, 1H), 2.04 (s, 3H).

13C NMR (150 MHz, D_2O) δ177.8, 135.9, 121.4, 107.4, 105.9, 105.6, 103.6, 103.0, 82.2, 81.3, 79.8, 78.1, 77.6, 77.5, 77.4, 77.2, 77.1, 75.5, 75.1, 74.7, 73.5, 73.4, 73.3, 73.2, 72.9, 71.6, 71.2, 70.6, 70.4, 70.2, 63.6, 63.6, 63.0, 62.9, 62.7, 54.1, 24.9

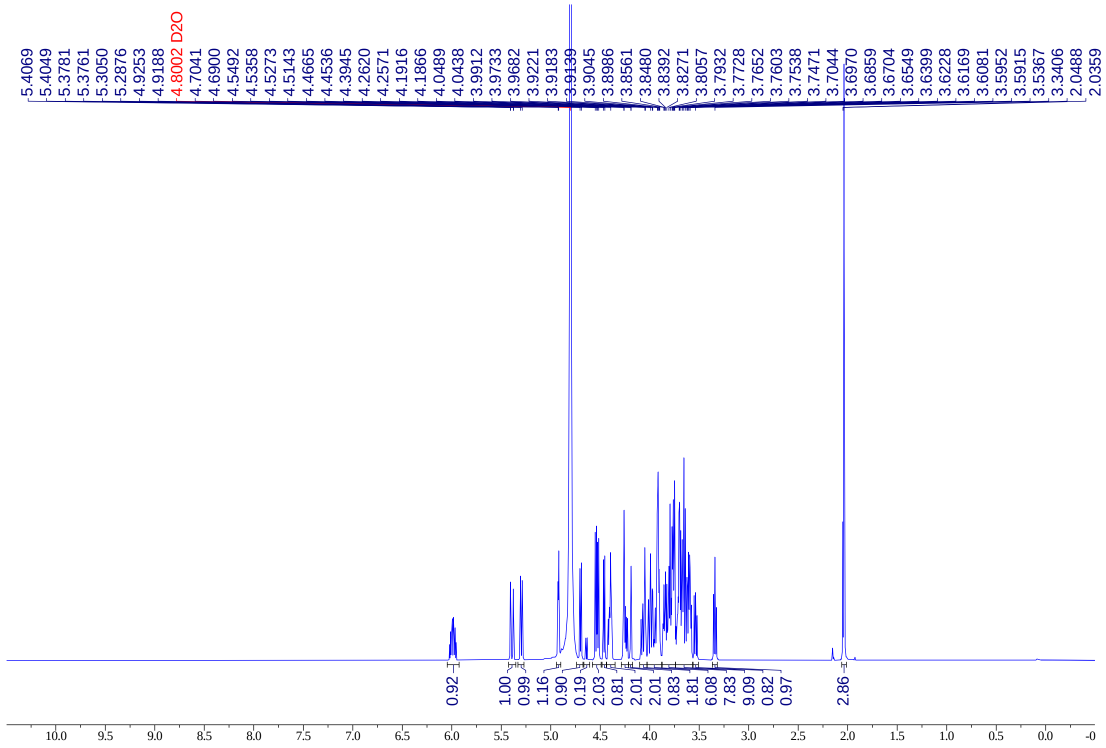

^{1}H NMR spectra of Allyl-Gb5

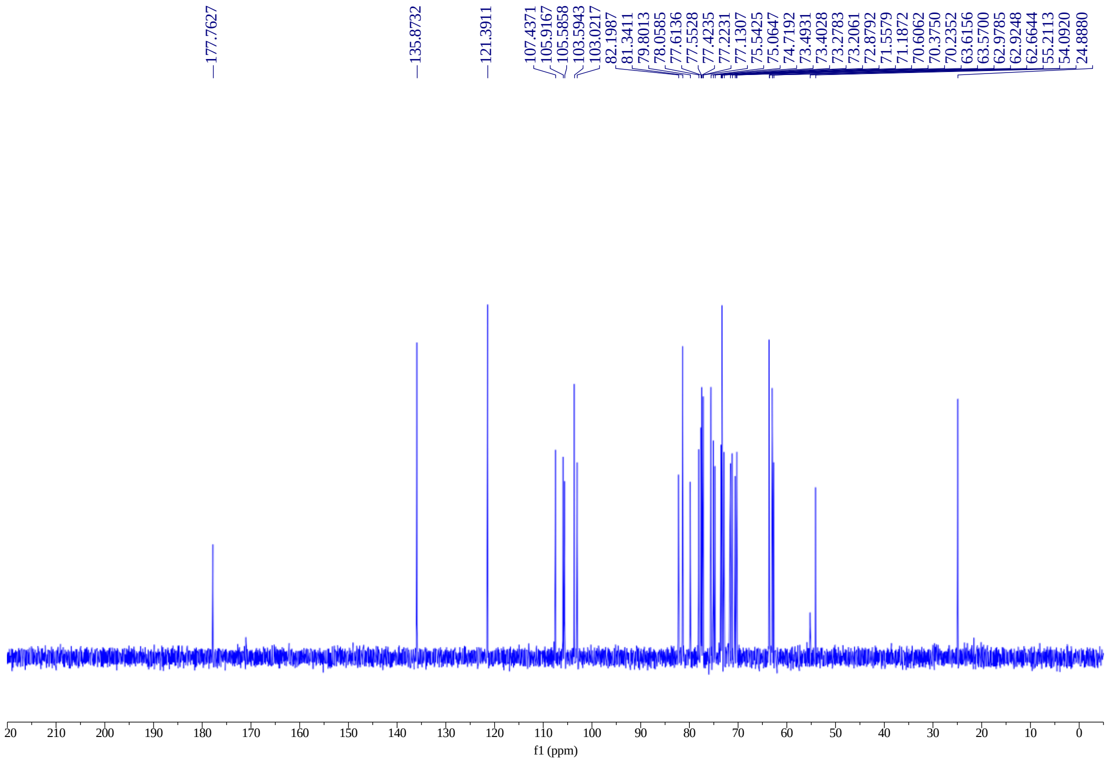

^{13}C NMR spectra of Allyl-Gb5

Gb5-C_5Cl

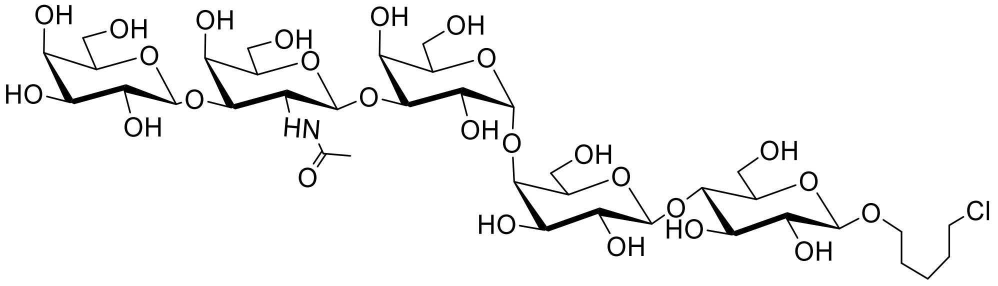

N-((2S,3R,4R,5R,6R)-2-(((2R,3R,4S,5S,6R)-2-(((2R,3R,4R,5R,6S)-6-(((2R,3S,4R,5R,6R)-6-((5-chloropentyl)oxy)-4,5-dihydroxy-2-(hydroxymethyl)tetrahydro-2H-pyran-3-yl)oxy)-4,5-dihydroxy-2-(hydroxymethyl)tetrahydro-2H-pyran-3-yl)oxy)-3,5-dihydroxy-6-(hydroxymethyl)tetrahydro-2H-pyran-4-yl)oxy)-5-hydroxy-6-(hydroxymethyl)-4-(((2R,3R,4S,5R,6R)-3,4,5-trihydroxy-6-(hydroxymethyl)tetrahydro-2H-pyran-2-yl)oxy)tetrahydro-2H-pyran-3-yl)acetamide

1H NMR (600 MHz, D_2O) δ4.92 (d, *J* = 4.0 Hz, 1H), 4.70 (d, *J* = 8.5 Hz, 1H), 4.52 (d, *J* = 7.7 Hz, 1H), 4.71 (d, *J* = 8.0 Hz, 1H), 4.46(d, *J* = 7.7 Hz, 1H), 4.40-4.38 (m, 1H), 4.26 (d, *J* = 2.9 Hz, 1H), 4.19 (d, *J* = 3.1 Hz, 1H), 4.09-3.90 (m, 9H), 3.86-3.58 (m, 20H), 3.55-3.52 (t, *J* = 7.8 Hz, 1H), 3.32-3.29 (t, *J* = 8.5 Hz, 1H), 2.03 (s, 3H), 1.84-1.79 (m, 2H), 1.96-1.64 (m, 2H), 1.54-1.50 (m, 2H).

13C NMR (150 MHz, D_2O) δ175.1, 104.8, 103.3, 102.9, 101.9, 100.4, 79.5, 78.7, 78.7, 77.2, 75.4, 75.0, 74.8, 74.6, 74.5, 72.9, 72.4, 72.1, 70.8, 70.6, 70.3, 70.2, 68.9, 68.5, 68.0, 67.6, 61.0, 60.9, 60.3, 60.3, 60.0, 51.4, 45.5, 31.5, 28.0, 22.5, 22.2.

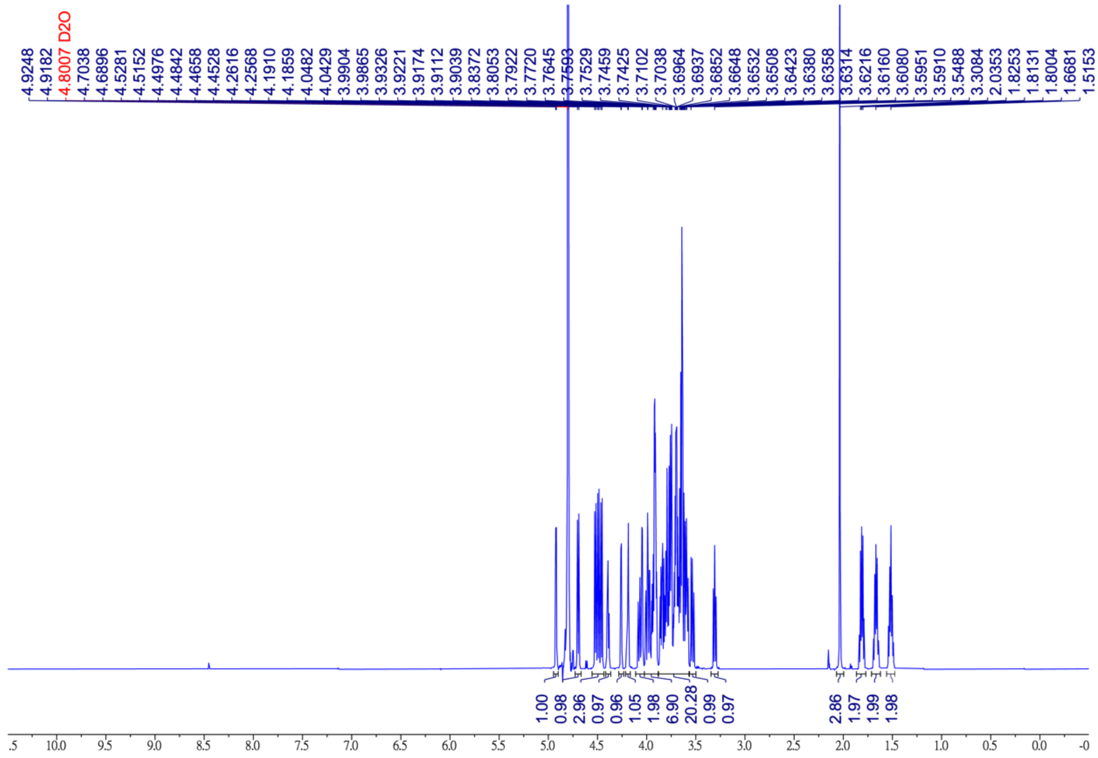

^{1}H NMR spectra of Gb5-C_5Cl

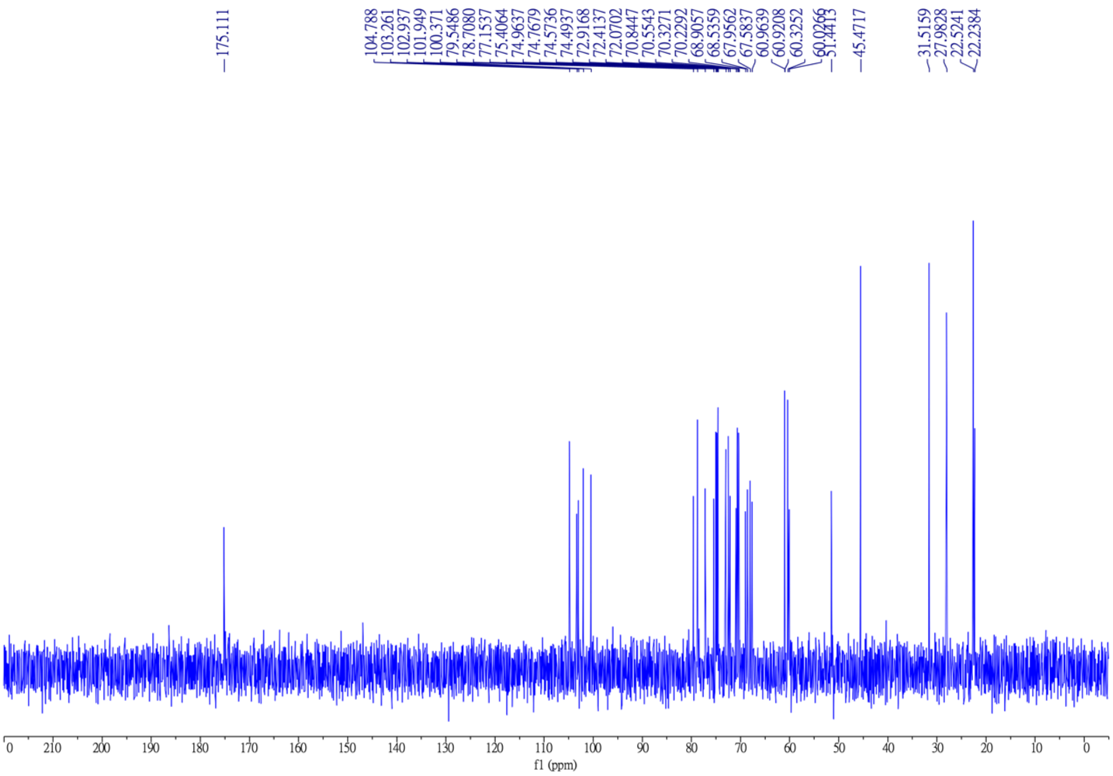

^{13}C NMR spectra of Gb5-C_5Cl

Gal-β1, 3-GlcNAc-β1, 3-Gal-OMe

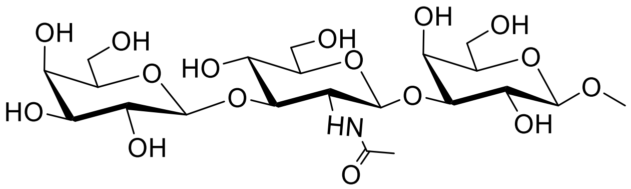

N-((2S,3R,4R,5R,6R)-2-(((2R,3S,4S,5R,6R)-3,5-dihydroxy-2-(hydroxymethyl)-6-methoxytetrahydro-2H-pyran-4-yl)oxy)-5-hydroxy-6-(hydroxymethyl)-4-(((2R,3R,4S,5R,6R)-3,4,5-trihydroxy-6-(hydroxymethyl)tetrahydro-2H-pyran-2-yl)oxy)tetrahydro-2H-pyran-3-yl)acetamide

1H NMR (600 MHz, D_2O) δ4.75 (d, *J* = 8.4 Hz, 1H), 4.44 (d, *J* = 7.7 Hz, 1H), 4.31 (d, *J* = 7.9 Hz, 1H), 4.14 (d, *J* = 3.2 Hz, 1H), 3.92-3.87(m, 3H), 3.84-3.68 (m, 9H),3.65-3.63 (dd, *J* = 3.3 Hz, 9.9 Hz 1H), 3.59-3.47 (m, 7H), 2.02 (s, 3H)

13C NMR (150 MHz, D_2O) δ177.8, 106.6, 106.2, 105.2, 84.9, 84.8, 78.0, 77.9, 77.4, 75.2, 73.4, 72.5, 71.2, 71.2, 71.1, 63.8, 63.6, 63.2, 59.9, 57.5, 25.0

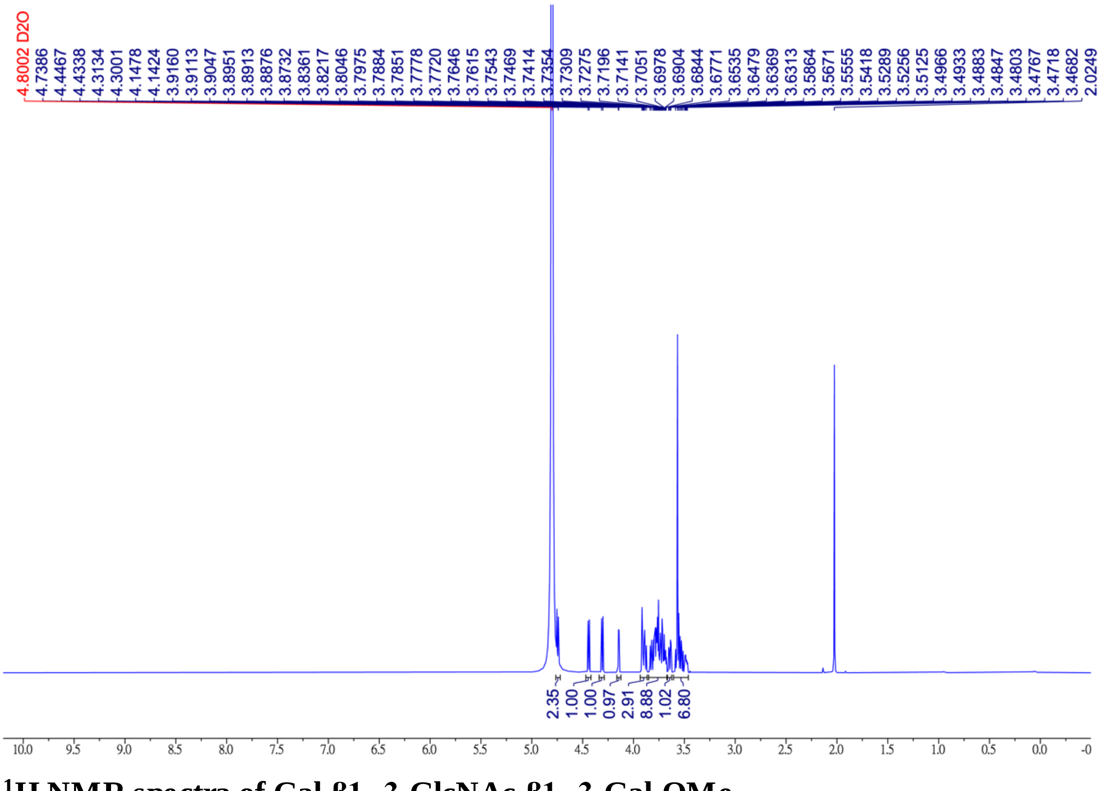

^{1}H NMR spectra of Gal-β1, 3-GlcNAc-β1, 3-Gal-OMe

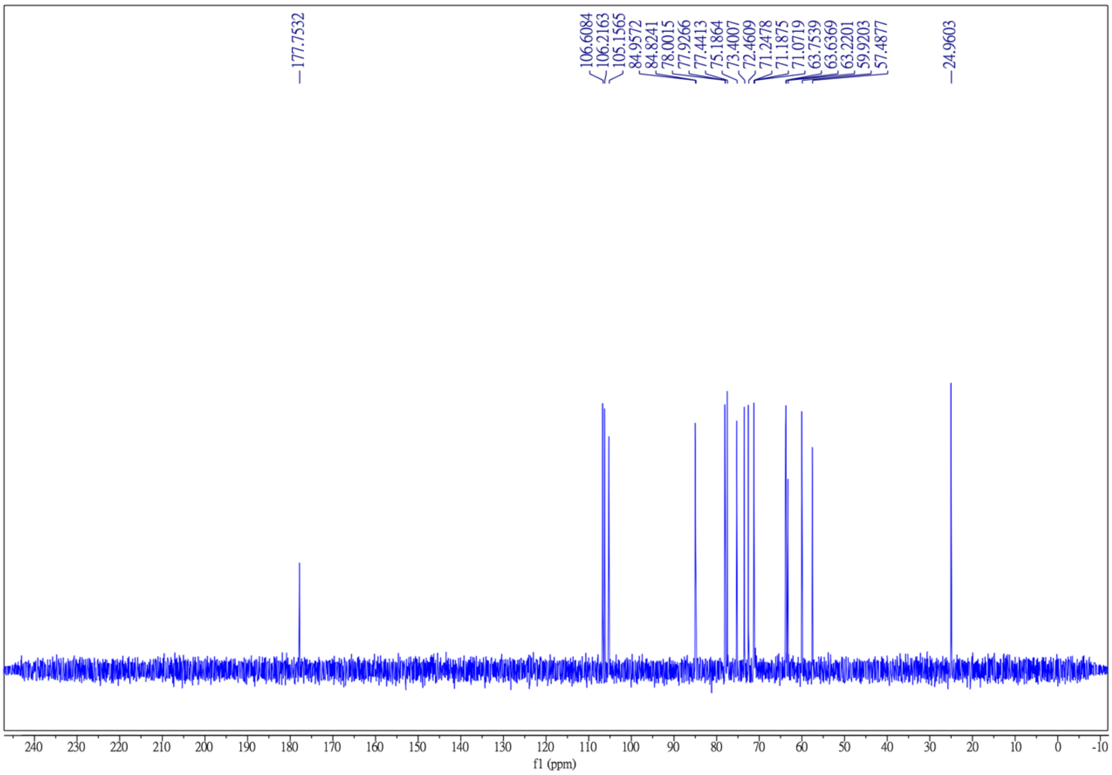

^{13}C NMR spectra of Gal-β1, 3-GlcNAc-β1, 3-Gal-OMe

Gal-β1, 3-Man-β1, 6-Man

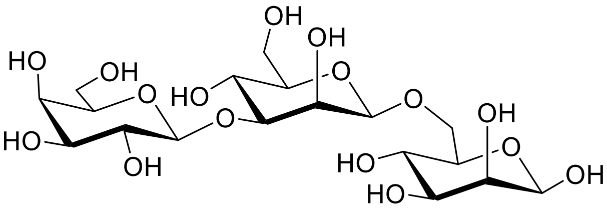

(2R,3S,4S,5S,6R)-6-((((2R,3S,4S,5R,6R)-3,5-dihydroxy-6-(hydroxymethyl)-4-(((2S,3R,4S,5R,6R)-3,4,5-trihydroxy-6-(hydroxymethyl)tetrahydro-2H-pyran-2-yl)oxy)tetrahydro-2H-pyran-2-yl)oxy)methyl)tetrahydro-2H-pyran-2,3,4,5-tetraol

1H NMR (600 MHz, D_2O) δ5.15 (d, J = 1.68Hz, 1H), 4.88 (d, J = 1.02Hz, 0.5H), 4.70 (d, J = 0.72 Hz, 0.5H), 4.69 (d, J = 0.66 Hz, 1H), 4.52 (d, J = 7.74 Hz, 2H), 4.24 (d, J = 2.34, 1H), 4.20-4.14 (m, 2H), 3.96-3.90 (m, 10H), 3.86-3.57 (m, 20H), 3.53-3.50 (m, 0.5H), 3.41-3.39 (m, 2H)

13C NMR (150 MHz, D_2O) δ 100.8, 100.3, 94.2, 93.8, 79.9, 76.0, 75.4, 75.0, 73.0, 72.6, 71.3, 71.2, 70.8, 70.6, 70.2, 68.7, 68.6, 68.1, 68.0, 66.7, 66.5, 65.3, 61.2, 61.1

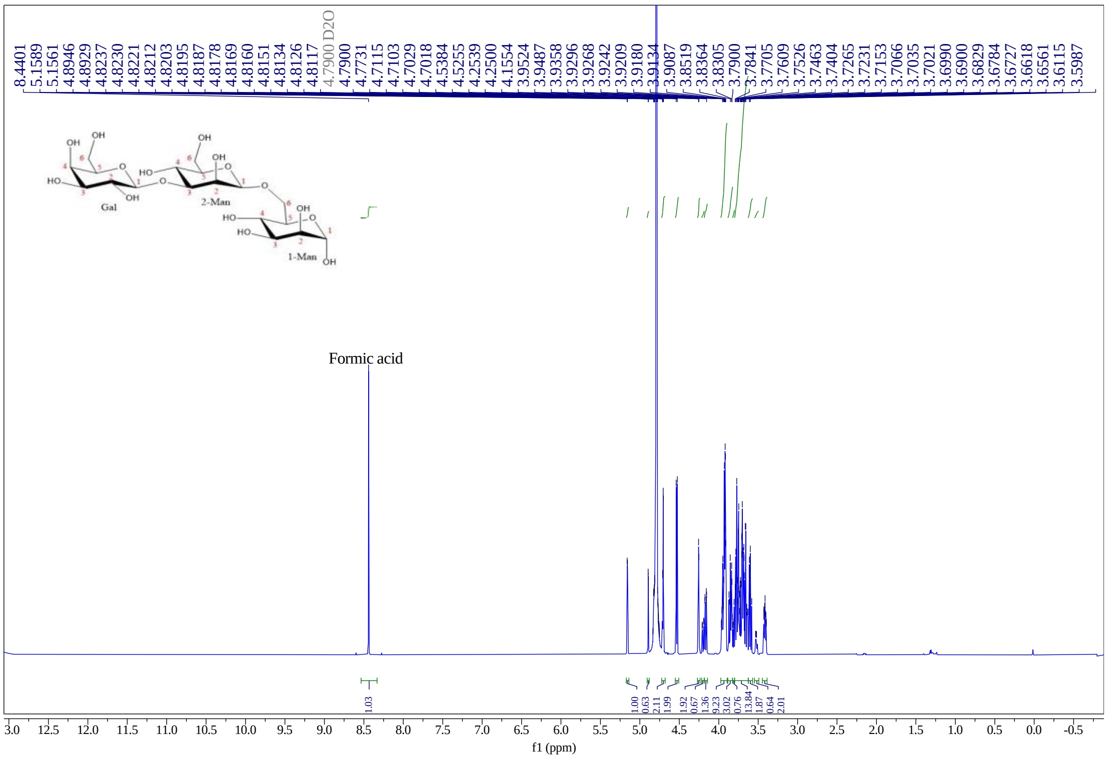

^{1}H NMR spectra of Gal-β1, 3-Man-β1, 6-Man

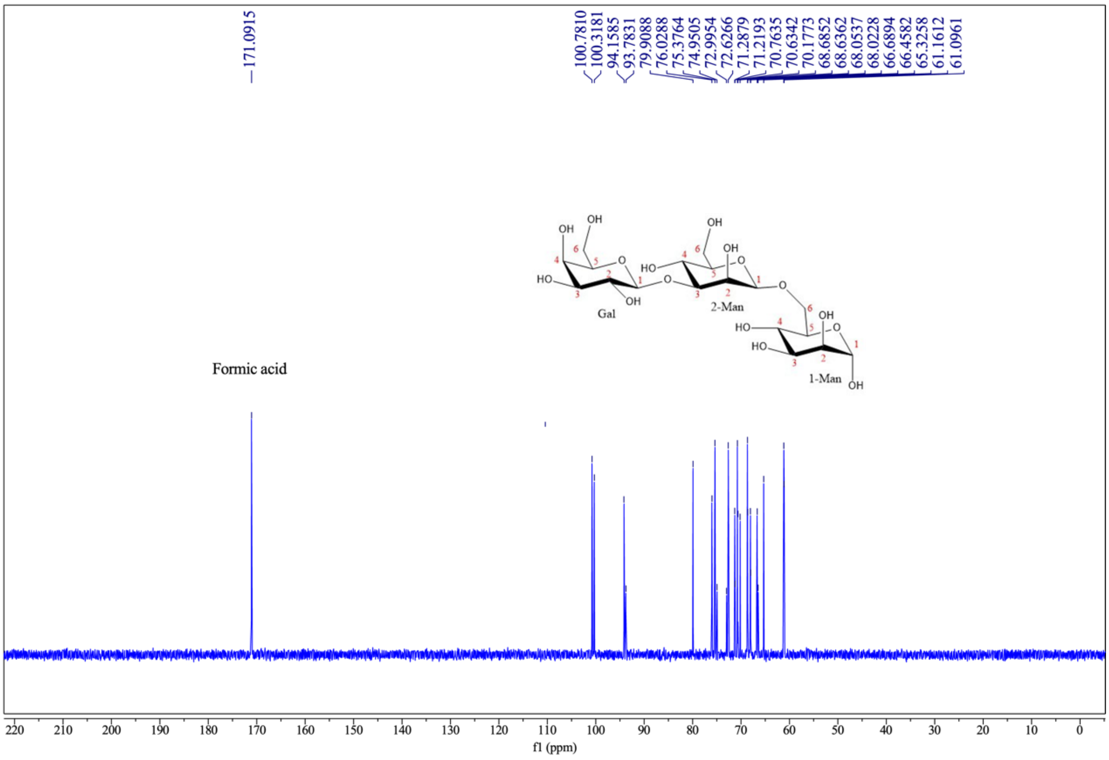

^{13}C NMR spectra of Gal-β1, 3-Man-β1, 6-Man

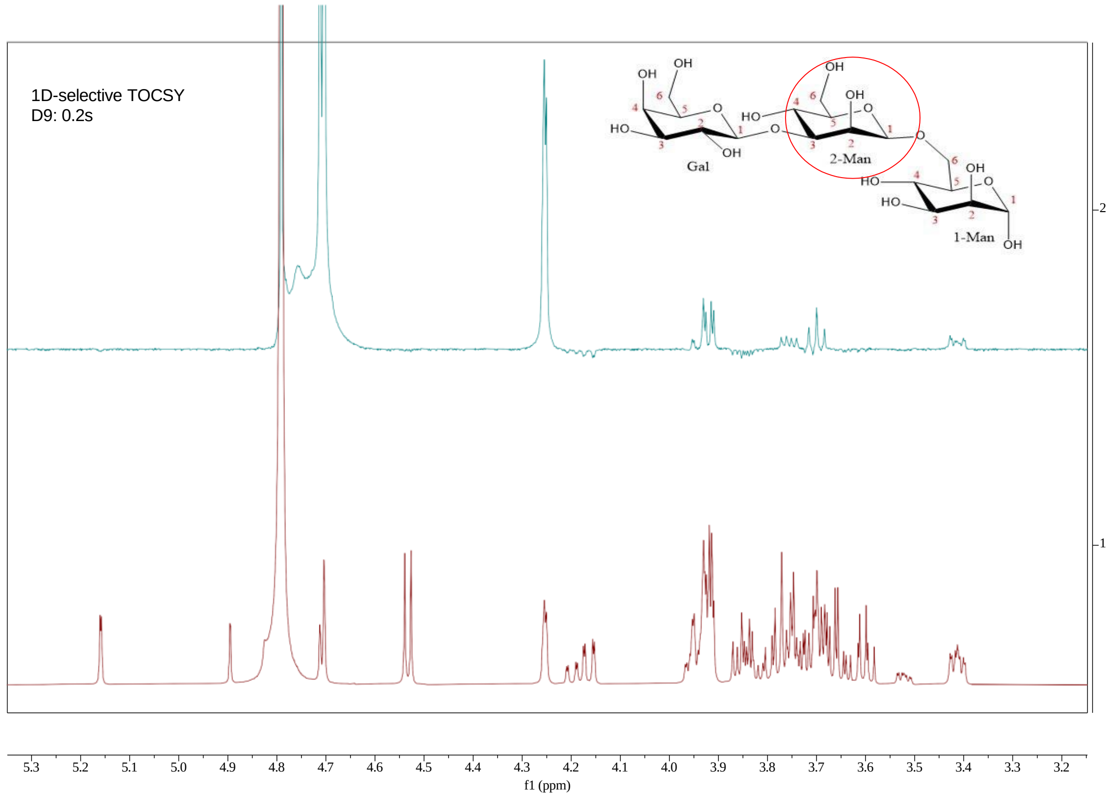

1D selective TOCSY spectra of Gal-β1, 3-Man-β1, 6-Man

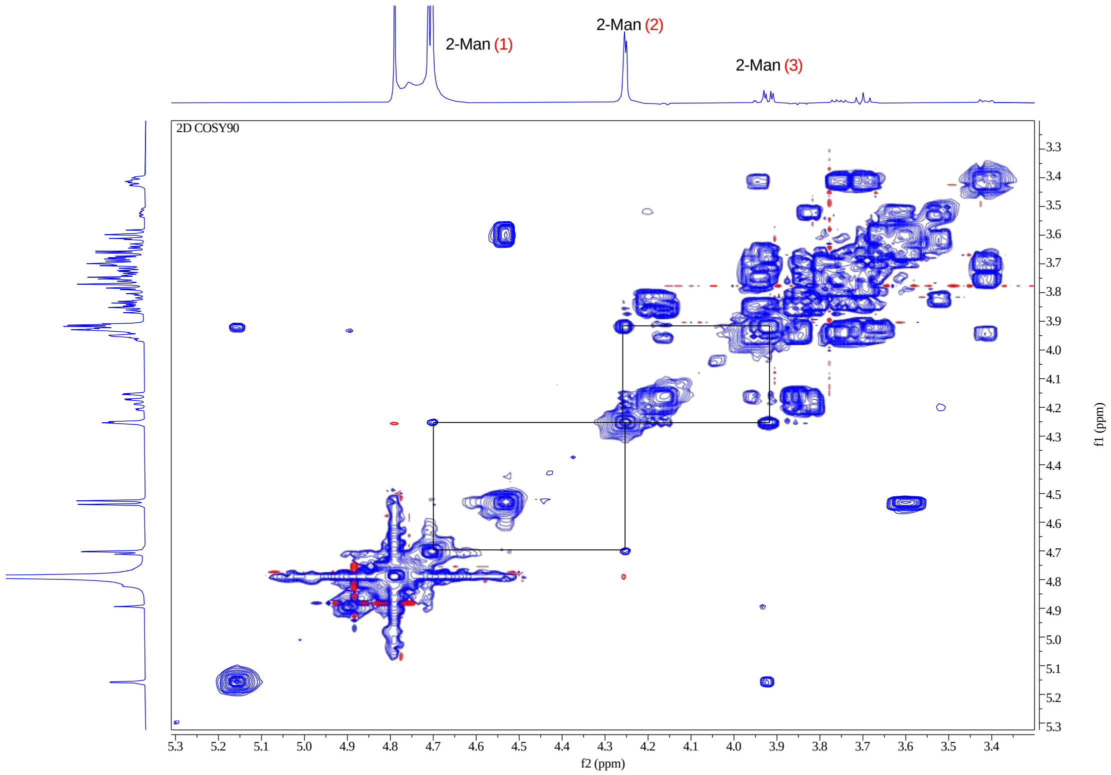

2D COSY90 spectra of Gal-β1, 3-Man-β1, 6-Man

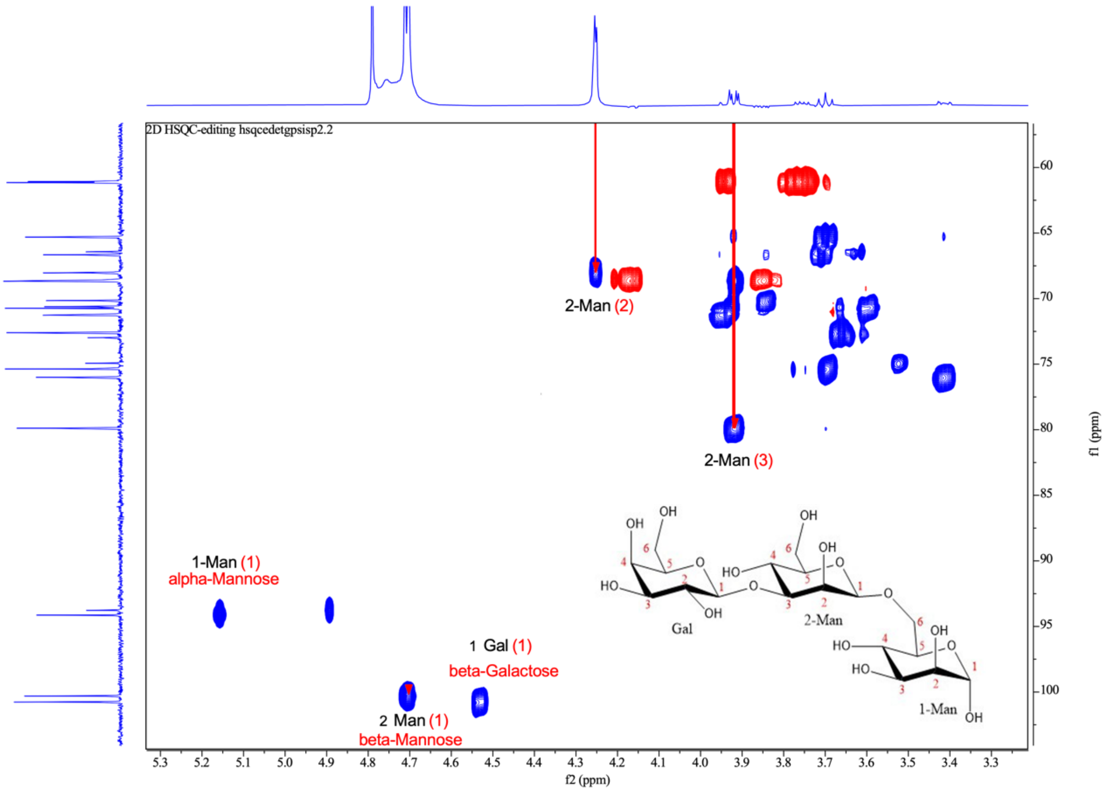

2D HSQC spectra of Gal-β1, 3-Man-β1, 6-Man

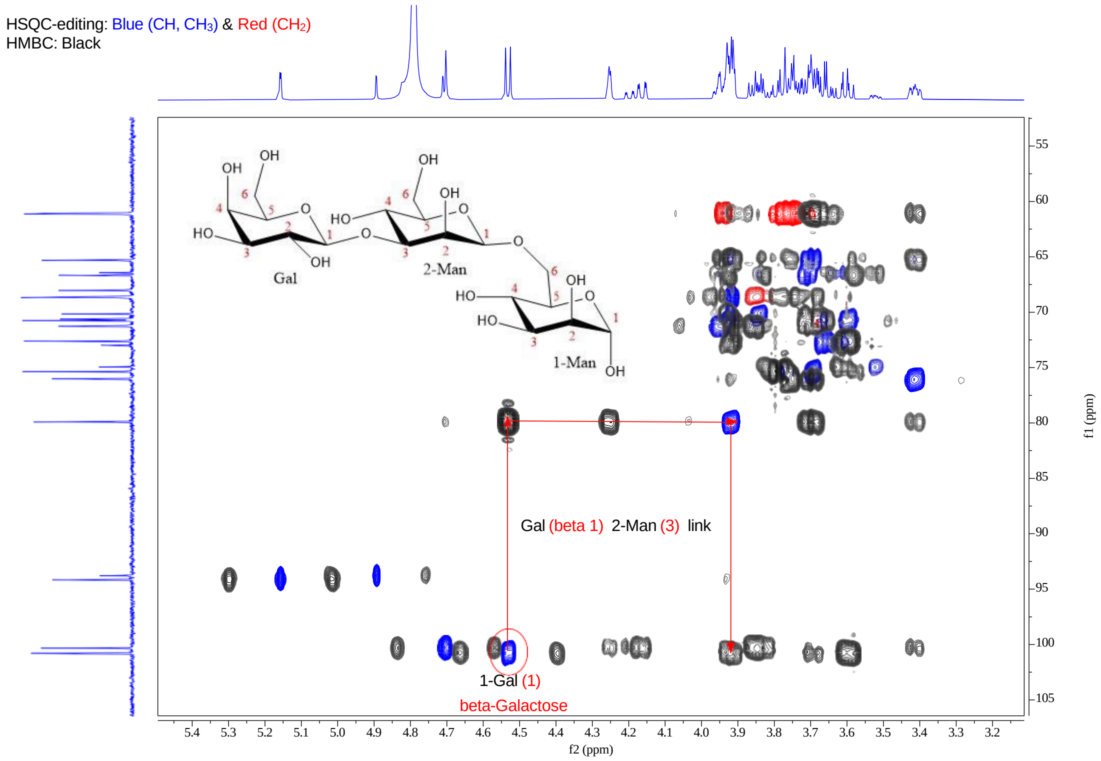

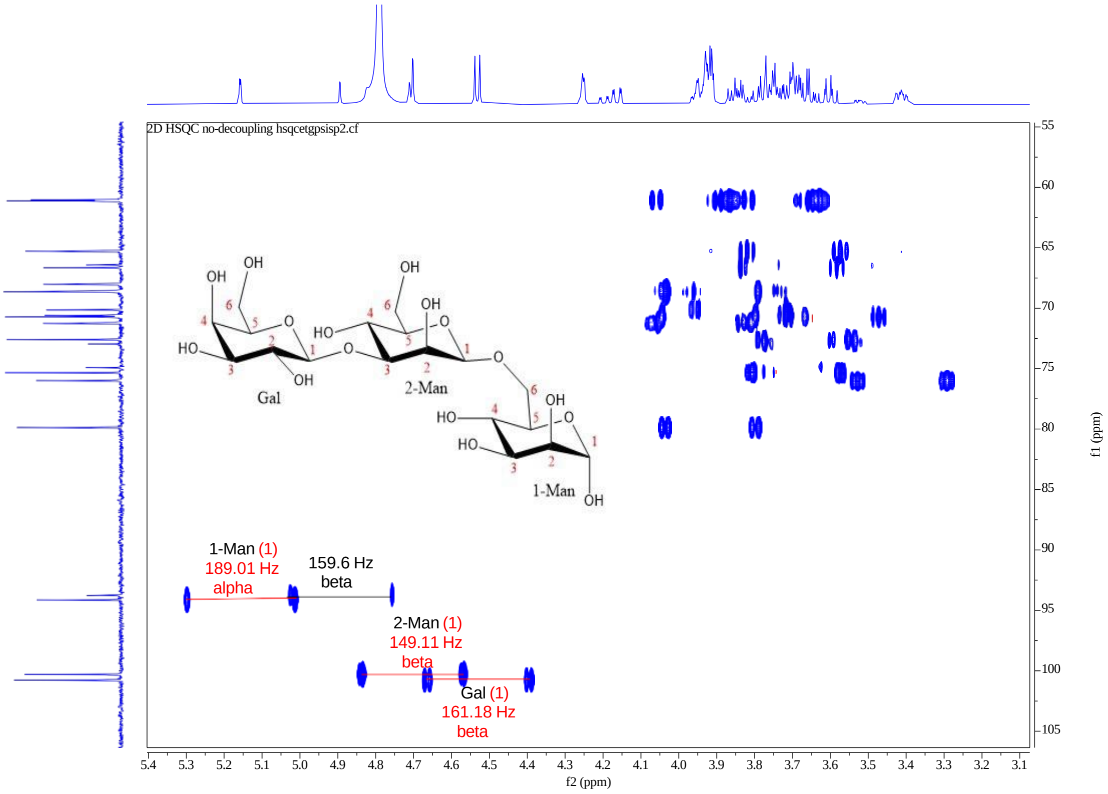

2D HSQC no-decoupling hsqcetgpsisp2.cf
Gal
2-Man
1-Man
1-Man (1)
189.01 Hz
alpha
159.6 Hz
beta
2-Man (1)
149.11 Hz
beta
Gal (1)
161.18 Hz
beta
f2 (ppm)
f1 (ppm)

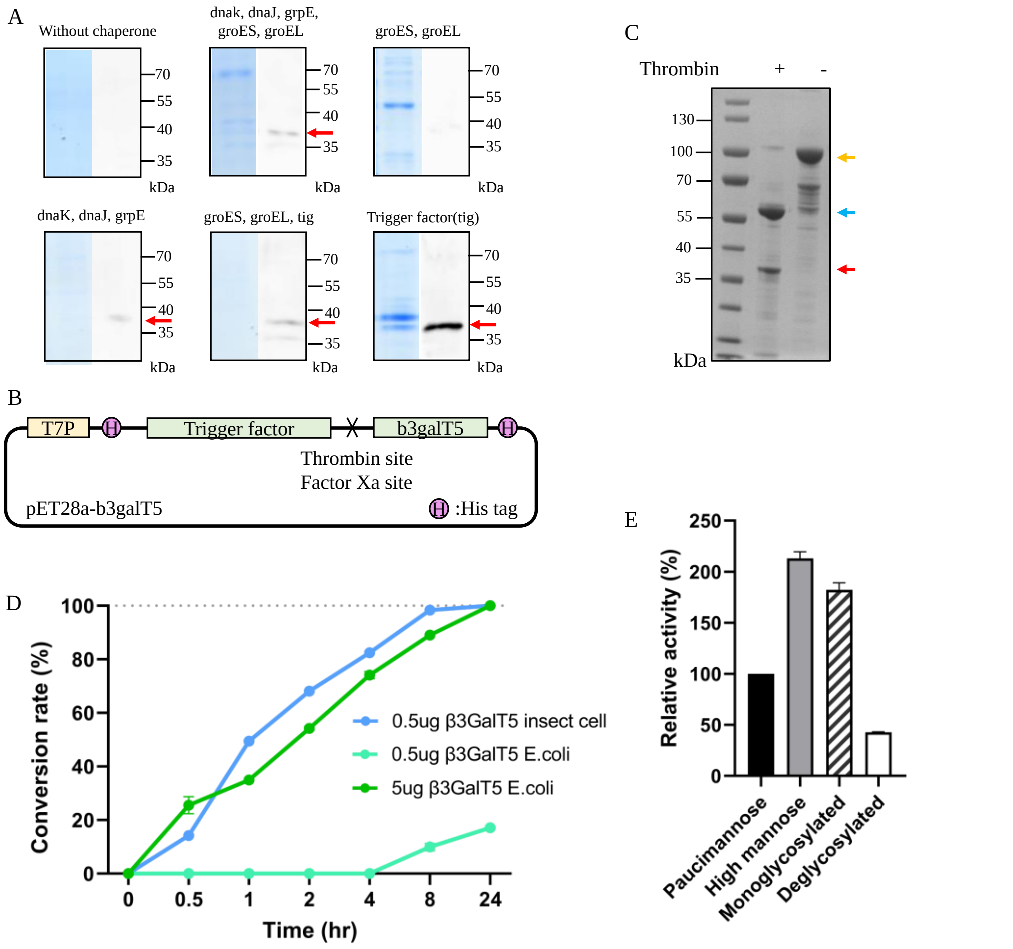

Figure S6: Expression of recombinant soluble β3GalT5-1 (N29-V310) fused with N-terminal molecular chaperone trigger factor (TF) in *E. coli*. (A) Recombinant soluble β3GalT5-1 was expressed in *E. coli* under various conditions: without molecular chaperone, with dnaK, dnaJ, grpE, groES, groEL, groES, and groEL, dnaK, dnaJ, and grpE, groES, groEL, and trigger factor (TF). The results in the left panel were visualized through SDS-PAGE followed by Coomassie blue staining, while the right panel showed the western blot detected with anti-β3GalT5 antibodies. The recombinant β3GalT5-1 is indicated by red arrows. (B) Design of recombinant TF-β3GalT5-1 with the thrombin cutting site positioned between β3GalT5-1 and the trigger factor. (C) Purified TF-β3GalT5 with or without thrombin treatment to remove the trigger factor. The results were visualized through SDS-PAGE followed by Coomassie blue staining. The yellow arrow indicated TF-β3GalT5, the blue arrow showed the trigger factor, and the red arrow was β3GalT5-1. (D) Conversion of Gb4 glycan labeled with 2-aminobenzamide (2-AB) to 2-AB-SSEA-3 glycan at different time points with β3GalT5-1 and the products were analyzed using UPLC. (E) The activity of β3GalT5-1 bearing different glycoforms. The β3GalT5-1 glycoforms with paucimannose were expressed from insect cells, and treatment of cell culture with kifunensine provided the high mannose glycoforms which were further treated with Endo-H to obtain

monoglycosylated (mono-GlcNAc) or with PNGase F to generate de-glycosylayed β3GalT5-1.

Table S3: Comparison of Activity Between TF-β3GalT5-1 (N29-V310) and LgtD.

Enzyme	Thrombin	Relative activity (%) *
TF-β3GalT5-1	-	107 ±8
	+	209 ±13
LgtD	-	100

* Based on microbial LgtD as reference, all reactions contain 0.1 mM UDP-galactose, 5 mM Gb4 glycan, and 12.5μM TF-β3GalT5 or LgtD expressed from *E. coli*. The results are presented as the mean of three biological replicates ± SEM.

Table S4. Optimized Synthesis of SSEA-3 Using Soluble β3GalT5-1.

Molar ratio galactose/ Allyl-Gb4 glycan*	Molar ratio Allyl-Gb4 glycan/soluble β3GalT5-1	Allyl-Gb4 glycan	Allyl-Gb5 glycan	Allyl-gal-Gb5 (side product)
0.8	8000	0.8%	97%	2.2%
1	8000	0.7%	97%	2.3%
1.2	4	0.7%	84.3%	15%
2	4	0.6%	79.1%	20.3%
3.8	4	1.3%	90.7%	8%

*Different ratios of galactose in UDP-Gal to Gb4 glycan were incubated with recombinant soluble β3GalT5 from insect cells through sugar-nucleotide regeneration and the reaction mixture was analyzed by LC-MS after 69 hours.

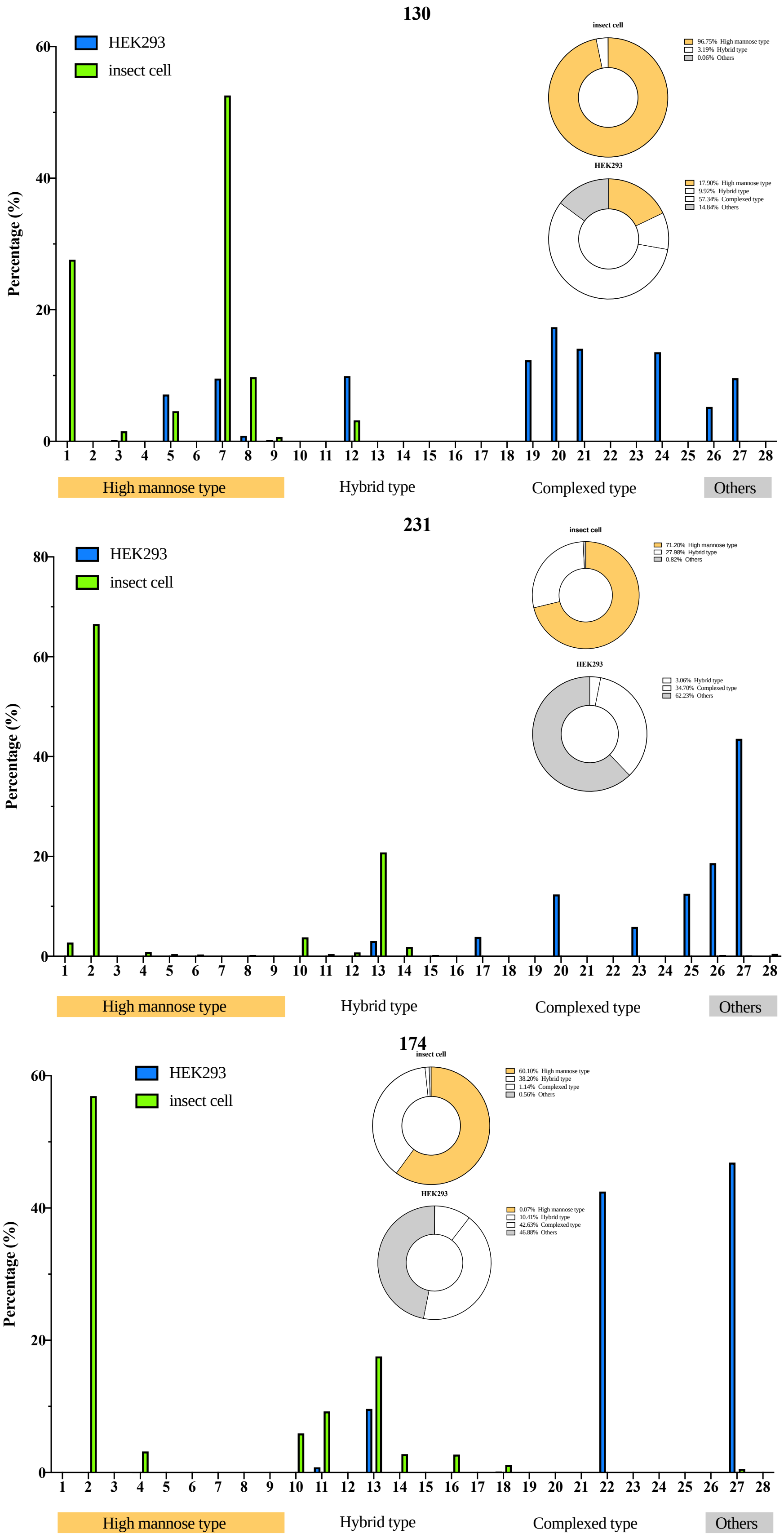

Figure S7: Compositions of the three N-glycosites (N-130, N-174, N-231) in β3GalT5-1 expressed from insect and mammalian cells. The symbol F stands for

fucose, S for sialic acid and the number after F/S stands for the number of fucose or sialic acid on glycans. For mannose type glycans, 1. Man3, 2. Man3F1 (paucimannose), 3. Man4, 4. Man4F1, 5. Man5, 6. Man5F1, 7. Man6, 8. Man7, 9. Man8; 10-16 as hybrid-type glycans; 17-25 as complex-type glycans; 26 as non-glycosylated; 27-28 as unclassified glycans.

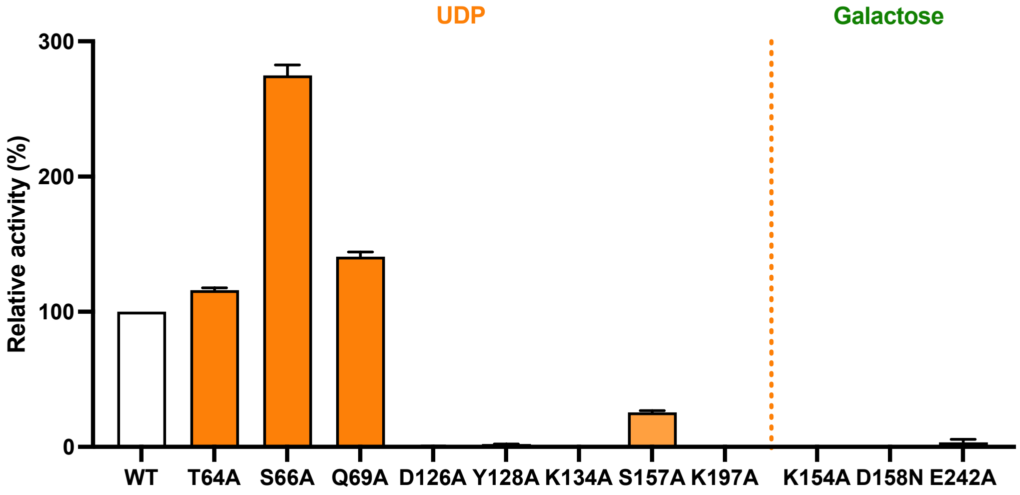

Figure S8. Alanine screening of the residues interacting with UDP-galactose. Residues involved in the binding of human β3GalT5-1 were mutated to alanine and the activities were assessed using an enzyme activity assay, with allyl-Gb4 glycan as the acceptor and UDP-galactose as donor. The orange color indicated the residues interacting with UDP, the green color represented the residues interacting with the galactose of UDP-galactose.

Table S5. Substrate specificity of soluble S66A β3GalT5-1

Substrate	Symbol	Relative activity (%)*
	GlcNAc and GalNAc as terminal end	
GlcNAc-β,3-Gal-OMe	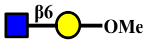	245 ±5
GalNAc-β,3-Gal	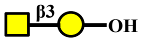	212 ±6
	Galactose and Mannose as terminal end	
Gal-β,6-GalNAc	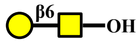	53 ±1
Gal-β,3-GalNAc	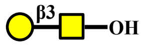	58 ±1
Gal-β,3-GalNAc-αOMe	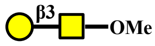	90 ±1
Gal-β,3-GalNAc-αO-Bn	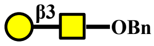	45 ±3
β-3Galactobiose	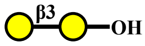	70 ±2
Gal-β-4-GalNAc	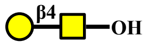	46 ±1
Lactose	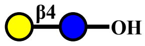	9 ±1
Gal-β-6-Gal	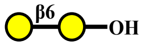	37 ±1
Man-α-1,6-Man	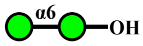	57 ±7
Man-β1,6-Man	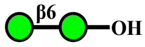	225 ±1
	Glycolipid and its derivatives	
Gb4 glycan	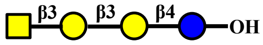	240 ±5
Gb4	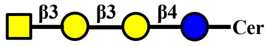	100
Gb4 glycan-C5Cl	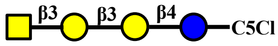	247 ±3
isoGb4 glycan-C5Cl	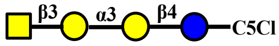	245 ±3
Lc3-glycan	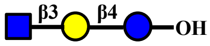	244 ±4

*Based on natural substrate Gb4 as reference, all reactions were performed in 20 mM HEPES (pH 7.4) buffer containing 0.1mM $MnCl_2$, 0.1 mM UDP-Gal, 5 mM substrate, and 5 μM soluble S66A β3GalT5-1 expressed from insect cells.

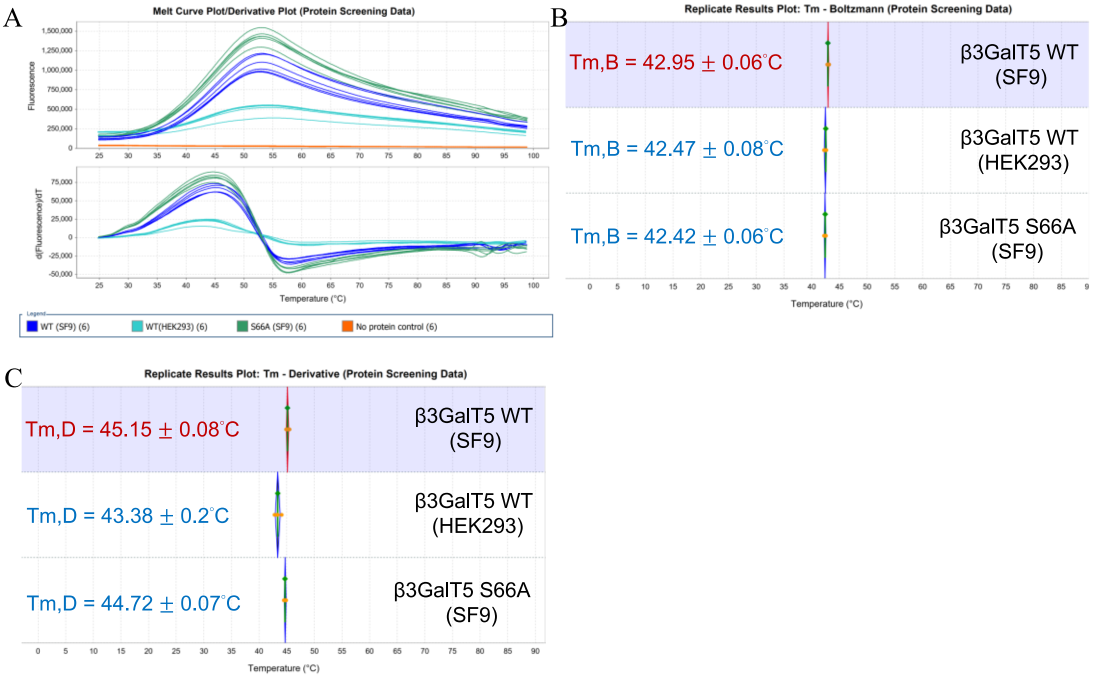

Figure S9. Thermostability of soluble β3GalT5-1 wild type (WT) and S66A mutant. (A) Melt curve plot of six replicates WT (expressed from HEK293, blue and insect cells, lake blue) and S66A (expressed from insect cells, green) soluble β3GalT5-1. (B)(C) Tm calculation by Boltzmann and Derivative methods.

Reference

(1) Tsai, T. I.; Lee, H. Y.; Chang, S. H.; Wang, C. H.; Tu, Y. C.; Lin, Y. C.; Hwang, D. R.; Wu, C. Y.; Wong, C.-H. Effective sugar nucleotide regeneration for the large-scale enzymatic synthesis of Globo H and SSEA4. *J Am Chem Soc* 2013, *135* (39), 14831-14839.

(2) Xia Y, Chu W, Qi Q, Xun L. New insights into the QuikChange™ process guide the use of Phusion DNA polymerase for site-directed mutagenesis. *Nucleic Acids Res.* 2015, 43(2):e12.

(3) Cheung, S. K.; Chuang, P. K.; Huang, H. W.; Hwang-Verslues, W. W.; Cho, C. H.; Yang, W. B.; Shen, C. N.; Hsiao, M.; Hsu, T. L.; Chang, C. F.; Wong, C.-H. Stage-specific embryonic antigen-3 (SSEA-3) and beta3GalT5 are cancer specific and significant markers for breast cancer stem cells. *Proc Natl Acad Sci U S A* **2016**, *113* (4), 960-965.
